# Fu’s subcutaneous needling approach versus electroacupuncture for knee osteoarthritis: protocol for comparative effectiveness and safety randomized controlled trial

**DOI:** 10.3389/fmed.2025.1510326

**Published:** 2025-05-29

**Authors:** Linan Liu, Jian Sun, Hu Li, Zhaojian Zheng, Jin Lu, Qin Lyu, Tianyu Bai

**Affiliations:** ^1^Shandong Provincial Third Hospital, Shandong University, Jinan, Shangdong, China; ^2^Graduate School of Shandong Sports College, Jinan, Shangdong, China; ^3^Clinical Medical College of Acupuncture Moxibustion and Rehabilitation, Guangzhou University of Chinese Medicine, Guangzhou, Guangdong, China; ^4^Shandong University of Traditional Chinese Medicine, Jinan, Shangdong, China; ^5^The First People’s Hospital of Wenling Bachelor’s degree, Wenling, Zhejiang, China; ^6^Nanjing Hospital of Chinese Medcine affiliated to Nanjing University of Chinese Medcine, Nanjing, Jiangsu, China

**Keywords:** Fu’s subcutaneous needling, electroacupuncture, knee osteoarthritis, protocol, effectiveness and safety, randomized controlled trial

## Abstract

**Introduction:**

With the aging of the population, knee osteoarthritis (KOA) is a common chronic osteoarthritic disorder among middle-aged and elderly people. Fu’s subcutaneous needling (FSN) and electroacupuncture (EA) are both effective means of treating KOA. FSN, with characterized by simple operation, quick effect, safety and green,is a modern acupuncture that integrates modern medicine with traditional acupuncture theory. Our team has demonstrated through clinical trials that FSN can effectively relieve pain and improve walking ability in patients with KOA. However, studies on the difference in clinical efficacy of FSN in treating KOA still lack clinical validation with large samples. The purpose of this study was to provide high-level clinical evidence for the clinical management of KOA by comparing the multicenter differences between FSN and EA in the treatment of this disease.

**Methods and analysis:**

This study is a prospective, multicenter, randomized controlled trial to compare the efficacy and safety of FSN with EA in the treatment of KOA. This study intends to recruit 300 patients with osteoarthritis of the knee in 6 tertiary hospitals in China, including Shandong Provincial Third Hospital, Shandong University, etc. The group was randomly divided into FSN group and EA group according to 1: 1 ratio. The intervention time is 30 min, and the treatment was carried out three times a week for 4 weeks, and the long-term follow-up was carried out one month after the treatment. The primary outcome index was the response rate of knee symptom relief, and the secondary outcome indexes were the numerical assessment of knee pain (NRS), WOMAC osteoarthritis index, quality of life evaluation (SF-12), 6-min walk test and safety evaluation.

**Clinical trial registration:**

ClinicalTrials.gov, ChiCTR2400080196.

## Highlights

- Firstly, this is a multicenter, large-sample study. It will provide strong evidence for the efficacy and safety of FSN in the treatment of KOA.
- Secondly, we will conduct this trial in strict compliance with the protocol in order to realistically evaluate the efficacy.
- Thirdly, blinding is not possible for both operators and participants.
- Fourthly, patients recruited from various centers may differ in gender, age, and other disorders, so subgroup analyses may be necessary to determine the effectiveness of FSN in different subgroups.
- Fifthly, this study did not have a sham-Fu’s subcutaneous needling group to remove the placebo effect.

## 1 Introduction

In a systematic analysis of the Global Burden of Disease Study, the number of KOA is predicted to increase by 74.9% (59.4–89.9) by 2050 compared to 2020 (1, 2). KOA is recognized as one of the 11th most disabling factors globally among 291 diseases (3). KOA, with degenerative changes of knee cartilage and secondary osteophytes, is a chronic osteoarthritic disorder (4). The main clinical manifestations are knee pain, localized tenderness with joint swelling, limited knee flexion and extension, and knee friction during activities. In severe cases, patients may suffer from the lack of mobility of the knee joint, which may lead to wasting muscle atrophy, and even symptoms such as knee inversion and knee valgus deformity (5). According statistics (6), the prevalence of KOA is 16%. It increases with age and seriously affects the quality of life of patients, placing a heavy burden on individuals, families and society (7). Therefore, early prevention and treatment are crucial for KOA patients.

The 2021 edition of the guidelines for osteoarthritis (8) points out that basic treatment methods including health education, functional exercise, and physical therapy are the preferred treatment modalities for patients. On this basis, topical NSAIDs (non-steroidal anti-inflammatory drugs) and oral NSAIDs are recommended as the first-line therapeutic drugs for KOA pain. Although medication can alleviate pain, it can be harmful to the human body, and patients may experience cardiovascular, gastrointestinal and other a series of adverse reactions (9). Surgery is expensive and traumatic, and many patients refuse it (10). These treatments are effective for KOA, but they have certain side effects, long treatment period and relatively high cost. Therefore, finding a safe and effective treatment for KOA with low recurrence rate and fewer side effects has become an urgent need for patients and doctors.

**Figure 1:**
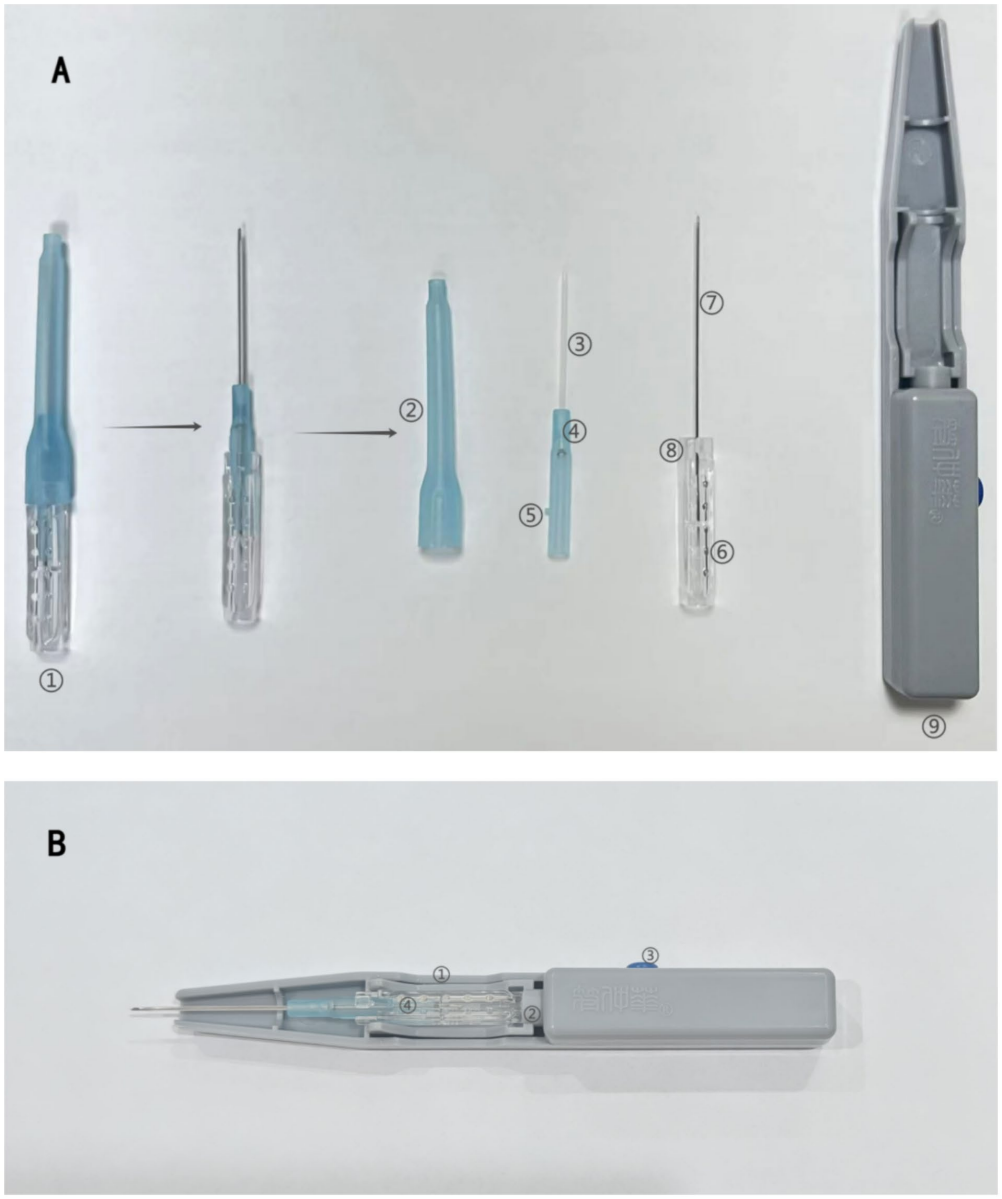
Structure of the Fu’s subcutaneous needling needle. **(A)** ① Fu’s subcutaneous needling. ② Protective cover. ③ Soft tube. ④ Built-in rivets. ⑤ Buckle upwards. ⑥ Point-like bulge. ⑦ Stainless steel needle. ⑧ Slot. ⑨ Inserting device. **(B)** ① Pedestals. ② Inserting device turning lever. ③ Control button. ④ Fu’s subcutaneous needling.

FSN is a needling activity that involves a sweeping maneuver in the subcutaneous tissue around or adjacent to the limbs of a confined ailment. FSN is, invented by Dr. Zhonghua Fu in 1996, a modern form of acupuncture that integrates modern basic medicine with traditional acupuncture theory (11). Compared with traditional acupuncture, FSN has distinctive characteristics due to its unique needles and needling method (Figure 1), and is effective in treating neck, shoulder, back, and limb pain due to a variety of causes (12, 13). FSN is to make the muscle as the therapeutic target, through sweeping and Reperfusion Approach (RA) eliminate the affected muscle, accelerate the circulation of local blood and lymphatic fluid, promote metabolism, so as to alleviate the muscle tension and spasm, thus restoring the muscle strength and coordination, increasing the mobility and stability of the joints, and promoting the rehabilitation of the knee joint function (14). There has been research evidence (11, 15, 16) that FSN can effectively improve pain and dysfunction in KOA, but there are few large sample studies comparing FSN with EA.

The purpose of this study was to compare the difference in clinical efficacy between FSN and EA for the treatment of KOA, and to provide high-level clinical evidence for the clinical treatment of KOA. Thus, the clinical efficacy of FSN treatment for KOA patients will be evaluated, and the results of the study will provide a new reference for the clinical treatment of KOA.

## 2 Methods and analysis

### 2.1 The design of study

In this study, 300 patients with KOA will be recruited in six tertiary hospitals in China, including Shandong Provincial Third Hospital, Shandong University, from January 2024 to June 2026, and randomly divided into the FSN group and the EA group, with 150 cases in each group, to validate the clinical efficacy of the multicenter and large sample.

Subjects will receive either FSN or EA treatment. Both groups will be treated for 4 weeks, with 3 treatments per week and 1 treatment every other day, for a total of 12 times. The primary outcome measure is the response rate of knee symptom relief after 4 weeks of treatment (the proportion of patients with a decrease of ≥2 points on the NRS and a decrease of ≥6 points on the WOMAC at the end of 4 weeks of treatment compared with baseline). Secondary outcome indicators is the response rate of knee symptom relief, NRS, WOMAC scale score, 6-min walk test at 1, 2, 3, and 4 weeks of intervention and 4, 12, and 24 weeks after the end of intervention, as well as the Brief Quality of Life Scale (SF-12) score at 1, 4, and 4 weeks after the end of intervention. The flow chart of this experiment is shown in Figure 1.

**Figure 2:**
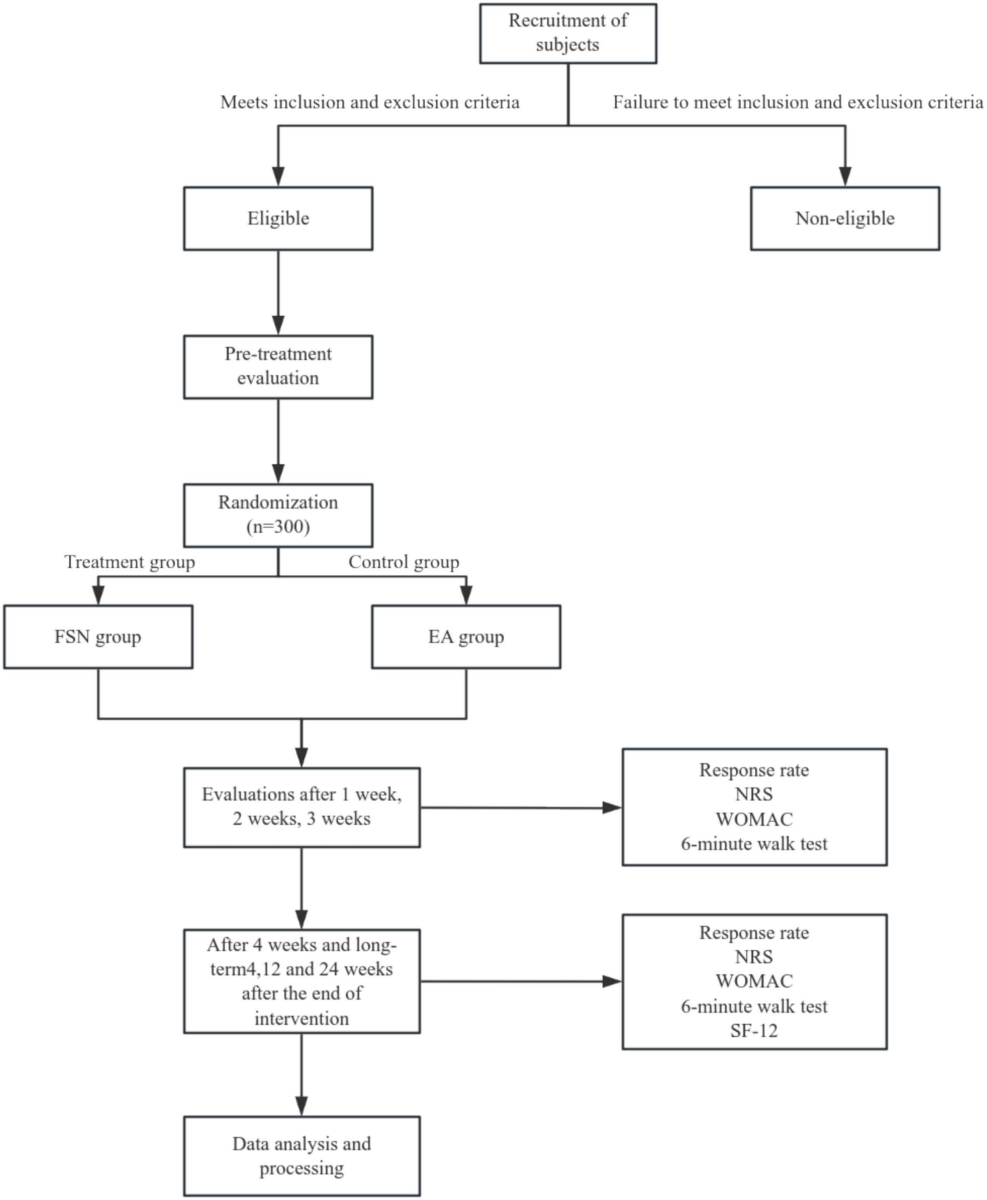
Trial profile over 4 weeks.

The study and experimental procedures were approved by the Institutional Review Board. All subjects will be recruited from 6 centers in the country (Figure 2).

## 3 Participants

### 3.1 Diagnostic standards

Referring to the diagnostic criteria for osteoarthritis of the knee in the Osteoarthritis Diagnostic and Treatment Guidelines (2018 Edition) formulated by the Joint Surgery Group of the Orthopedic Branch of the Chinese Medical Association (17).

(1) Recurrent episodes of knee pain in the past month.
(2) Age ≥50 years.
(3) Joint stiffness in the morning not higher than 30 min.
(4) Bone friction sound during knee joint movement.
(5) X-ray (standing or weight-bearing position) showing narrowing of the joint space, sclerosis and/or cystic degeneration of the chondral bone, and formation of bone capillaries at the joint margins.

Knee Osteoarthritis can be diagnosed if 1 and any 2 of 2, 3, 4, 5 are met.

### 3.2 Eligibility criteria

(1) Meets diagnostic criteria.
(2) Age≤ 75 years old, regardless of gender.
(3) Mean knee pain level NRS score ≥4 in the 2 weeks before enrollment.
(4) Classification of the condition, KOA imaging classification (Kellgren Lawrence, KL) gradeIIto III.
(5) Have not received acupuncture, herbal medicine, or other treatments in the last 1 month.
(6) Agree to participate in this study and sign the informed consent form.
(7) The above 6 items were met simultaneously to be included in the study.

### 3.3 Inclusion criteria

(1) Combined with serious cardiovascular, cerebrovascular, digestive, respiratory, urinary, hematopoietic system and other diseases.
(2) Knee pain caused by other diseases such as gouty arthritis, rheumatoid arthritis, and knee deformity.
(3) History of surgery, joint cavity injection, or severe trauma to the target knee within the last 1 year.
(4) Presence of contraindications to MRI examination, e.g., pacemakers, metal implants, fear of confined spaces, pregnancy preparation, pregnancy, etc.
(5) Those who refuse to sign the informed consent form.

### 3.4 Sample recruitment

In this study, 300 subjects will be recruited in 6 hospitals, namely Shandong Provincial Third Hospital, Shandong University, Tai’an Traditional Chinese Medicine Hospital, Guangdong Provincial Hospital of Traditional Chinese Medicine, Nanjing Hospital of Traditional Chinese Medicine, the Second Affiliated Hospital of Bengbu Medical College, and the First People’s Hospital of Wenling City, Zhejiang Province.

After eligible patients sign an informed consent form, statisticians will randomly assign subjects to either the treatment group or the control group according to block group randomization, with 150 subjects in each group. The control group will be treated with EA and the treatment group will be treated with FSN.

### 3.5 Sample size calculation

This study is a randomized controlled trial design, the experimental group is the Fu’s subcutaneous needling group, the control group is the acupuncture group, the response rate of the research subjects is the main evaluation index of observation, according to the review of the literature/pre-experimental results, the response rate of the experimental group p1 is 0.56, and the response rate of the control group p2 is 0.45, set the bilaterally *α* = 0.05 that is, unilateral 0.025, the degree of certainty that is, the 1 - *β* is 0.9, and the sample size ratio of the experimental group to the control group is 1:1, the noninferiority threshold is-0.1. The ratio of the sample size of the test group to that of the control group is 1:1, and the non-inferiority cut-off value is-0.1. Referring to the method of Chow et al. (18), the sample size of the test group is 118 cases and the sample size of the control group is 118 cases by using the R language calculation. Considering 20% of lost visits as well as refusal of visits, at least 146 cases in the experimental group and 146 cases in the control group were eventually needed, totaling a sample size of 300 cases for inclusion.

## 4 Randomization and blinding

Researchers will screen patients for eligibility and recruiters will be responsible for recruiting 300 individuals. Random numbers generated using Stata V.14.0 will be packaged and hidden in envelopes by a third-party statistician according to the principles of block randomization.

Due to the physical specificity of FSN treatment and EA treatment, it was not possible to blind participants and subjects. However, the researchers responsible for data collection from participants and statistical analysis will be unaware of the allocation in order to minimize bias in the reporting of subjective results.

## 5 Intervention methods

### 5.1 Electroacupuncture group

**Figure 3:**
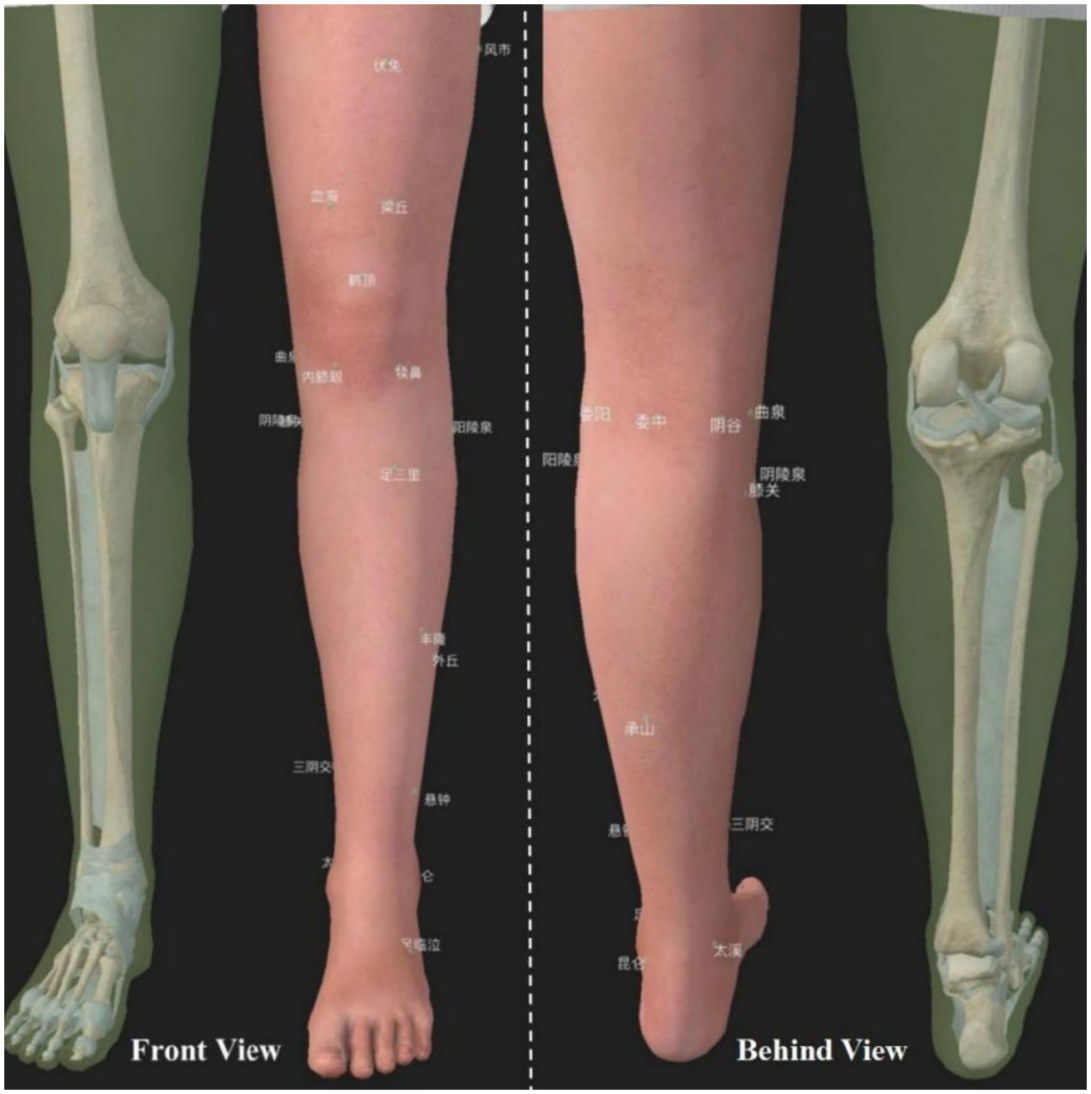
EA treatment points. 犊鼻: Du Bi (ST35), 内膝眼: Nei Xi-Yan (EX), 曲泉: Qu Quan (LR8), 膝阳关: Xi Yang-Guan (GB33), 伏兔: Fu Tu (ST32), 梁丘: Liang Qiu (ST34), 足三里: Zu San-Li (ST36), 丰隆: Feng Long (ST40), 鹤顶: He Ding (EX), 血海: Xue Hai (SP10), 阴谷: Yin Gu (KI10), 阴陵泉: Yin Ling-Quan (SP9), 三阴交: San Yin-Jiao (SP6), 太溪: Tai Xi (KI3), 风市: Feng Shi (GB31), 阳陵泉: Yang Ling-Quan (GB34), 外丘: Wai Qiu (GB36), 悬钟: Xuan Zhong (GB39), 足临泣: Zu Lin-Qi (GB41), 委阳: Wei Yang (BL39), 委中: Wei Zhong (BL40), 承山: Cheng Shan (BL57), 昆仑: Kun Lun (BL60).

Disposable Warren brand sterile acupuncture needles of 0.30mmx25mm or 0.30mmx40mm length were used, depending on the depth of the acupuncture point. EA treatment points (Table 1; Figure 3) will be referred to the study published by Prof. Liu Cunzhi in 2021 (19), including 5 necessary points, Du Bi (ST35), Nei Xi-Yan (EX), Qu Quan (LR8), Xi Yang-Guan (GB33), and A Shi point; According to different meridian diseases, 3 optional acupoints were selected, Foot Yangming meridian: Fu Tu (ST32), Liang Qiu (ST34), Zu San-Li (ST36), Feng Long (ST40), He Ding (EX); Foot Taiyin Spleen meridian: Xue Hai (SP10), Yin Gu (KI10), Yin Ling-Quan (SP9), San Yin-Jiao (SP6), Tai Xi (KI3), etc.;Foot ShaoYang Gallbladder meridian: Feng Shi (GB31), Yang Ling-Quan (GB34), Wai Qiu (GB36), Xuan Zhong (GB39), Zu Lin-Qi (GB41), etc.; Foot Solar Urinary Bladder meridian: Wei Yang (BL39), Wei Zhong (BL40), Cheng Shan (BL57), Kun Lun (BL60), etc. One group of EA was connected to Qu Quan/Xi Yang-Guan, and another group of EA was connected to the other two optional acupoints, with a frequency of 1 HZ, wave type of continuous wave, and current intensity to the extent that slight contraction of the muscles occurred; each treatment will last for 30 min, and the treatment will was performed 3 times per week, once every other day, for a total of 12 treatments in 4 weeks.

**Table 1:** EA treatment points.

Acupoint	Acupoint number	Acupoint position
Necessary Acupoints		
Du Bi	ST35	In the anterior knee region, in the lateral depression of the patellar ligament
Nei Xi-Yan	EX	At the knee, in the center of the medial depression of the patellar ligament
Qu Quan	LR8	In the knee, at the inner end of the popliteal crease, in the concave inner edge of the semitendinosus tendon
Xi Yang-Guan	GB33	In the depression between the knee, the posterior upper edge of the lateral epicondyle of the femur, and the tendon of the biceps femoris muscle and the iliotibial tract
A Shi point		Pain is the acupoint, and if there is pain, it is the acupoint
Optional Acupoints		
Fu Tu	ST32	At the anterior femoral region, six inches above the patellar floor, at the line connecting the anterior superior iliac spine and the lateral end of the patellar floor.
Liang Qiu	ST34	In the anterior femoral area, 2 inches above the patellar floor, between the lateral femoral muscle and the tendon of the rectus femoris muscle
Zu San-Li	ST36	On the outer side of the calf, 3 inches below the calf’s nose, 1 transverse finger outside the anterior tibial spine
Feng Long	ST40	On the outer side of the calf, 8 inches above the tip of the outer ankle, at the outer edge of the anterior tibial muscle, at a transverse finger on the outer side of the Tiaokou
He Ding	EX	In the anterior knee region, in the depression above the midpoint of the patellar floor
Xue Hai	SP10	In the anterior femoral area, 2 inches above the medial end of the patella, at the bulging point of the medial femoral muscle
Yin Ling-Quan	SP9	In the depression between the medial condylar edge of the tibia and the medial edge of the tibia on the inner side of the calf
Yin Gu	KI10	Posterior to the knee, on the transverse popliteal stripe, lateral margin of the semitendinosus tendon
San Yin-Jiao	SP6	On the inner side of the calf, 3 inches above the tip of the inner ankle, at the posterior edge of the medial edge of the tibia
Tai Xi	KI3	In the ankle area, the depression between the inner ankle tip and the Achilles’tendon
Feng Shi	GB31	In the femur, 7 inches above the patellar floor: with the hand upright and the palm pressed against the thigh, the depression indicated by the middle finger tip and the posterior edge of the iliotibial tract
Yang Ling-Quan	GB34	On the outer side of the calf, in the depression below the anterior part of the fibular head
Wai Qiu	GB36	On the outer side of the calf, 7 inches above the outer ankle tip, at the anterior edge of the fibula
Xuan Zhong	GB39	On the outer side of the calf, 3 inches above the outer ankle tip, at the anterior edge of the fibula
Zu Lin-Qi	GB41	On the dorsum of the foot, in front of the junction of the 4th and 5th metatarsal bones, in the lateral depression of the extensor digitorum longus tendon of the 5th toe
Wei Yang	BL39	At the knee, along the popliteal crease, at the inner edge of the biceps femoris tendon
Wei Zhong	BL40	In the posterior knee area, at the midpoint of the popliteal crease
Cheng Shan	BL57	At the intersection of the gastrocnemius abdomens and tendons in the posterior region of the calf
Kun Lun	BL60	In the depression between the outer ankle tip and the Achilles tendon in the ankle area

### 5.2 Fu’s subcutaneous needling group

**Figure 4:**
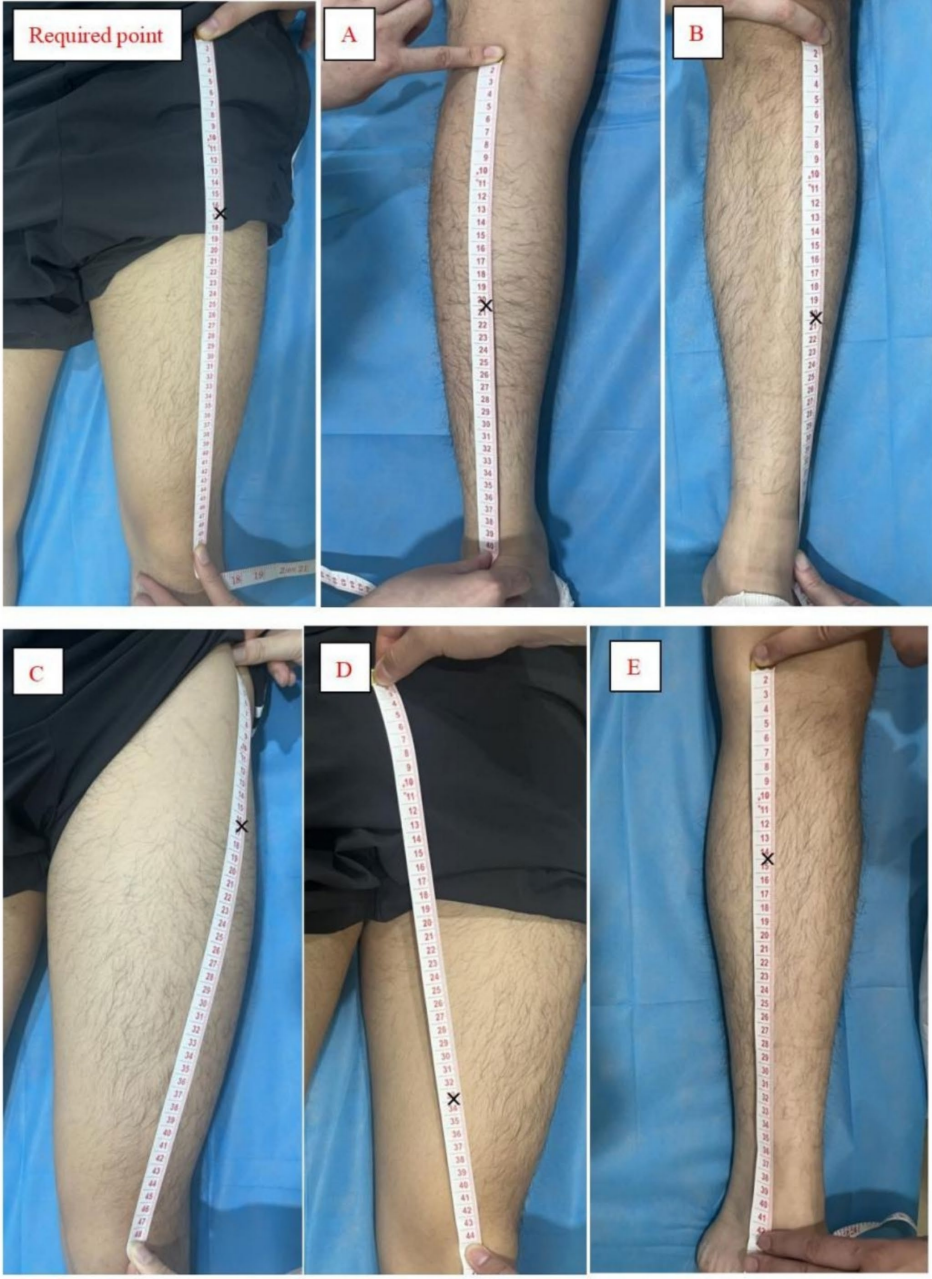
FSN treatment points. According to the soft ruler as a size reference, × represents the position of the needle entry point of the FSN. **(A)** For pain in the posterior side of the knee, choose the midpoint of the line connecting the popliteal fossa and the heel. For lateral knee pain, choose **(B)** the lateral border of the tibia, at the midpoint of the line between the external knee eye and the tip of the external ankle, or **(C)** the upper 1/3 of the line between the highest point of the greater trochanter of the femur and the upper outer edge of the patella. For medial knee pain, choose **(D)** the lower 1/4 of the line connecting the midpoint of the pubic symphysis with the inner superior border of the patella, or **(E)** the upper 1/3 of the line connecting the tip of the inner medial ankle of the lower leg with the medial tibial condyle.

**Figure 5:**
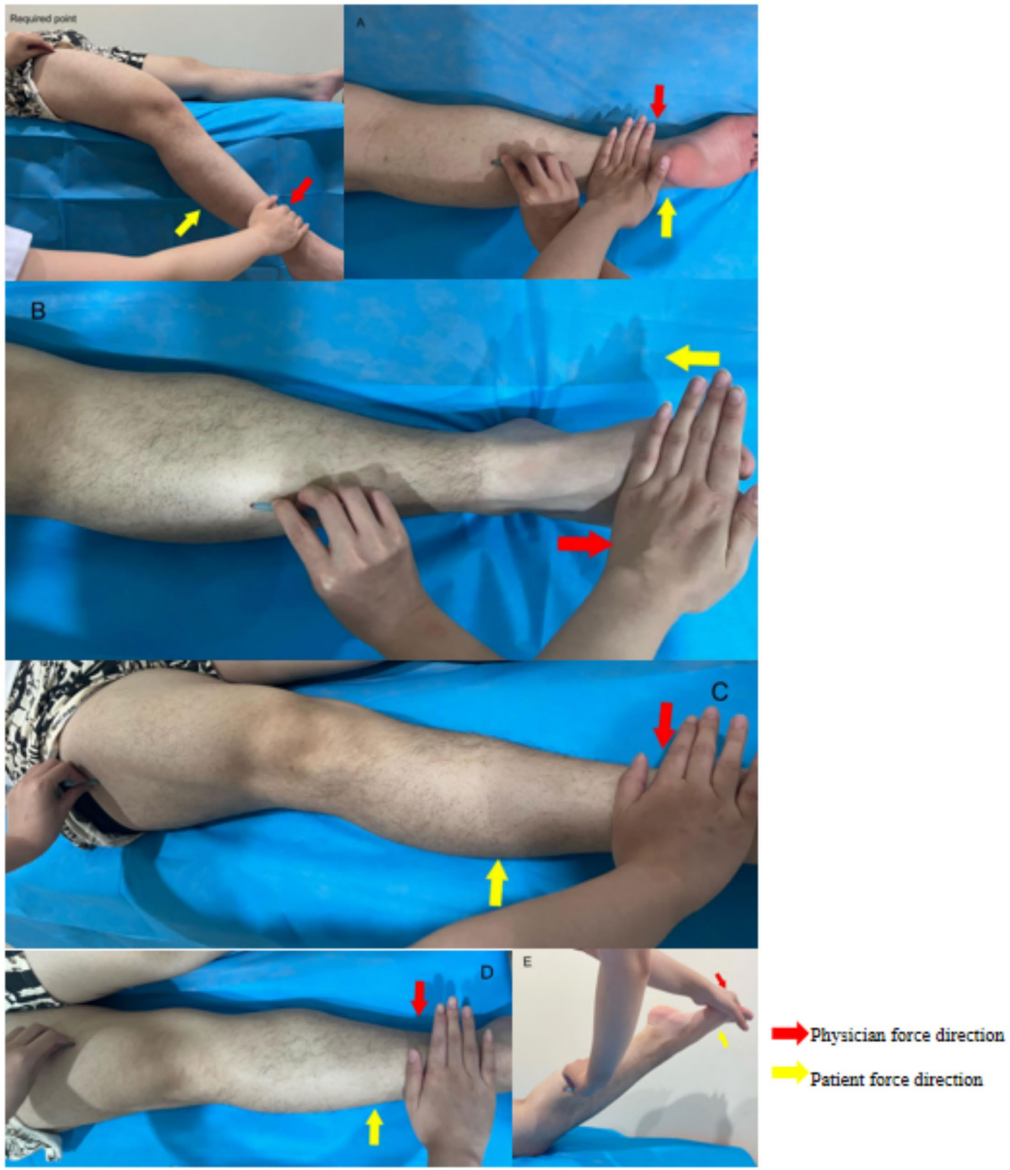
**(A–E)** Patient force and physician’s resistance in the reperfusion approach.

The disposable FSN produced by Nanjing Paifu Medical Technology Co. (Figure 1), and the specific operation will be as follows:

(1) Body position: the subject will be asked to lie supine with knees straight and pelvis in neutral position.
(2) Choose the needle entry points (Figure 4): Including 1 necessary point, which is a reference to previous studies (15, 20), the quadriceps muscle is selected and the entry point is between the line connecting the anterior superior iliac spine and the upper 1/3 of the lateral superior border of the patella. According to different pain sites, clinical manifestations and the finding of the affected muscle, choose 1 to 3 optional points among the following points of needle entry: A for pain in the posterior side of the knee, choose the midpoint of the line connecting the popliteal fossa and the heel. For lateral knee pain, choose B the lateral border of the tibia, at the midpoint of the line between the external knee eye and the tip of the external ankle, or C the upper 1/3 of the line between the highest point of the greater trochanter of the femur and the upper outer edge of the patella. For medial knee pain, choose D the lower 1/4 of the line connecting the midpoint of the pubic symphysis with the inner superior border of the patella, or E the upper 1/3 of the line connecting the tip of the inner medial ankle of the lower leg with the medial tibial condyle.
(3) Needle insertion: After selecting the needle insertion point, the therapist carries out routine disinfection, and then selects a disposable medium-gauge FSN (produced by Nanjing Paifu Medical Science and Technology Co., Ltd.), which is placed in the FSN insertion device (produced by Nanjing Paifu Medical Science and Technology Co., Ltd.), with the tip of the needle exposed outside of the flexible cannula, and the tip of the needle inserted in the direction of the knee joint, rapidly entering the subcutaneous layer of loose connective tissue around the affected muscle until the needle is completely immersed in the subcutaneous tissue. To confirm that the needle does not extend into the dermis or muscle, the participant must confirm that there is no pain at all during the entire insertion process.
(4) Swaying movement: The handle of the needle is held in the hand and the needle is swung from side to side in a fan shape. The fan angle is approximately 60°, and the swaying movement time for each needle entry point is 2 min, 200 times. Throughout the swaying movement, participants must confirm that they feel no pain at all.
(5) Reperfusion Approach (RA): the subject was required to do 3 cycles of RA for each movement, each cycle included 10 s of continuous movement and 10 s of rest, totaling 1 min, the specific RA is shown in Figure 5.
1 RA of mandatory points: the patient was placed in supine position, and the subject was asked to dispose of the popliteal fossa of the affected leg at the edge of the bed, with the calf suspended perpendicular to the treatment bed, and the therapist applied a resistance at the patient’s ankle, and the patient was instructed to straighten the knee joint.
2 RA at point A: the patient is positioned prone, the treatment staff applies a resistance at the patient’s ankle, and the subject is asked to flex the knee.
3 RA at point B: With the patient in the supine position, the therapist applies a resistance at the dorsum of the patient’s foot and asks the subject to perform dorsiflexion of the foot.
4 RA at point C: The patient is placed in the supine position and the therapist applies a resistance at the patient’s ankle and asks the subject to perform knee extension.
5 RA at point D: The patient is placed in the supine position and the therapist applies a resistance at the patient’s ankles and asks the subject to perform knee extensions.
6 RA at point E: With the patient in the prone position, the patient is instructed to perform a knee flexion maneuver and the therapist applies a resistance at the plantar aspect of the patient’s foot and asks the subject to perform a plantarflexion maneuver.

The therapist performs the reperfusion activity with the corresponding movements according to the position of the performed points.

(6) Withdrawal of needles: At the end of the treatment, remove the FSN.

## 6 Outcomes measurements

Evaluators will be tested for outcomes at baseline, week 1, week 2, week 3, week 4 of treatment, and during follow-up (Table 2).

**Table 2:** Study schedule.

Time point	Study period						
	Screening	Baseline	Treatment				Follow- up
	(−T1)	(T0)	(T1)	(T2)	(T3)	(T4)	(T8)
Basic information collection							
Eligibility screen	×						
Informed consent	×						
Allocation		×					
Demographic data		×					
Medical and treatment history		×					
Knee joint X-ray		×					
Safety observation							
Adverse event			×	×	×	×	
Therapeutic observation							
Level walking (NRS)		×	×	×	×	×	×
Stair activity (NRS)		×	×	×	×	×	×
6-min walk test		×	×	×	×	×	×
WOMAC		×	×	×	×	×	×
SF-12		×				×	×

−T1, the day before enrolment. T0, the day of enrolment and allocation. T1–T4, the treatment period for 4 weeks. T8, the Fourth week after the end of treatment. NRS, Numerical Rating Scale; WOMAC, the Western Ontario and Mc Master University Osteoarthritis Index; SF-12, Quality of Life Evaluation Scale.

### 6.1 Primary outcome

Response rate for knee symptom relief after 4 weeks of treatment (proportion of patients with ≥2-point decrease in joint pain NRS and ≥6-point decrease in WOMAC at the end of 4 weeks of treatment compared with baseline).

### 6.2 Secondary outcomes

Response rates for knee symptom relief, knee pain NRS scores, WOMAC scale scores, 6-min walk test, and Brief Quality of Life Scale (SF-12) scores at 1, 2, and 3 weeks of intervention and 4 weeks after the end of intervention.

### 6.3 Safety evaluation

Adverse events related to FSN and needling occurred during the study period, including subcutaneous hemorrhage, needle fainting, needle breakage, etc., will be recorded, as well as the entry point of FSN selected for each treatment and the acupoints selected for electroacupuncture.

### 6.4 Patient and public involvement statement

Patients and the public will not be involved.

## 7 Statistical analysis

Statistical analysis was performed by SPSS26.0 software, and all test data were expressed as mean and standard deviation.

A total of two types of independent variables were used in this study, namely the group independent variable (FSN group and EA group) and the time independent variable (pre-intervention, after 1, 2, 3, and 4 weeks of intervention, and after follow-up), in which the time independent variable was a replicated independent variable. Data were tested for normality using the Shapiro–Wilk test and Leven’s test for chi-square. If the data conformed to normal distribution, two-way repeated measures ANOVA (Two-Way Repeated ANOVA) was used to observe the Main Effect and Interaction of the two types of independent variables, i.e., time independent variable (within-group) and subgroup independent variable (between-groups), on the dependent variable, and when there was an Interaction between the two, a Boneferroni’s *post hoc* test (*Post Hoc*) was used. η^2^ was used for the expression of the effect size: η^2^ < 0.06 is a small effect size. 0.06 ≤ η^2^ < 0.14 is a medium effect size. η^2^ ≥ 0.14 is a large effect size. If the distribution did not conform to normal distribution, the Scheirer-Ray-Hare test was used and results were expressed as median and interquartile spacing.

## 8 Ethics and dissemination

The protocol was registered in the ClinicalTrials.gov Registry (No. ChiCTR2400080196) and was ethically approved by the Ethics Committee of Shandong Provincial Third Hospital, Shandong University (approval number: KYLL-2023092). The Institutional Ethics Committee of Shandong Provincial Third Hospital, Shandong University will be responsible for the safety and quality control of this study, and all researchers will comply with medical ethics during the trial. Before enrollment, patients will be fully informed about the clinical trial and sign an informed consent form. For CRF, confirmation of the completion of each patient’s name, randomization number, gender, intervention plan assignment, adverse events, and outcome indicators is required. All relevant patient information will be kept strictly secret. Data accessibility will require approval from an independent ethics committee 12 months after publication. Other researchers can request datasets by emailing the corresponding author. The results of this study will be published in peer-reviewed journals or as a paper.

## 9 Discussion

Currently, the pathogenesis of KOA is not clear, and it is characterized by high morbidity and disability (2, 21). Relieving pain, slowing down the progression of the disease, improving the ability to walk, and improving the quality of life are the fundamental objectives of treating this disease (22).

EA is widely recognized internationally as an effective treatment for KOA. For example, the Chinese Orthopedic Association and the American College of Rheumatology (8, 23) have recommended the use of EA in the treatment of KOA, which can improve the clinical symptoms and quality of life of KOA patients (19). Many studies have shown that EA can regulate the expression of related cytokines, inhibit the apoptosis of chondrocytes, the degradation of cartilage extracellular matrix, and the generation of osteoclasts. In addition, EA can promote the proliferation of chondrocytes and cartilage repair, and play a bidirectional role in regulating the cartilage tissue, thus improving the patient’s knee joint lesions, and enhancing the clinical therapeutic effect (24). Moreover, In 2022 the UK NICE Association published a new version of its osteoarthritis guideline, which, based largely on the evidence from high-quality clinical studies by Prof. Cunzhi Liu’s team, concluded that electroacupuncture is clinically beneficial and cost-effective, and recommended the use of EA for the treatment of KOA (25). Evidently, EA therapy is mature and has been internationally recognized.

FSN is an emerging physical therapy in recent years, which is characterized by simple operation, fast effect, safety and green (26). It can sweep and pull the loose connective tissues to produce piezoelectricity and antipiezoelectricity effect, which breaks the energy crisis of the local affected muscles, and then cooperate with the reperfusion activities to relax the muscles and accelerate the blood circulation, so as to achieve the purpose of relieving the pain (27). In addition, the needling site of FSN is not at acupoints but at the normal tissues surrounding the myofascial excitation pain point (MTrP), which acts at the subcutaneous level of loose connective tissue (12, 28). It has been shown that (29) stretching has a direct mechanical effect on the inflammation-regulating molecules in connective tissues, and that when mechanical strain is produced by external forces, the crystals become electrodepolarized or electrically fielded, generating a positive piezoelectric effect; the channels of loose connective tissues are composed of proteins and mucopolysaccharides with semiconducting properties, and themselves contain a large amount of body fluids, and therefore transmit information up to three times faster than in the nervous system (30, 31). However, there is a lack of large sample clinical studies on the improvement of KOA by FSN. The results of this study will investigate whether FSN can be a viable and effective treatment.

The mandatory point of needle insertion in this study was selected with reference to the article published by Zhonghua Fu’s team (11, 15), which was the upper 1/3 of the line connecting the anterior superior iliac spine and the lateral superior edge of the patella. The optional points for needle insertion were selected based on clinical experience, according to the subjects’ different pain sites, clinical manifestations, and the location of the affected muscles, and 1–3 optional points were selected for the gastrocnemius muscle, tibialis anterior muscle, rectus femoris muscle, suture muscle, and soleus muscle.

The primary outcome is the response rate (32), which was defined as the proportion of participants who achieved minimal clinically important improvement (MCII) on both the NRS and the WOMAC functional scale. Minimal Clinically Important Improvement (MCII) is the smallest change in a patient’s symptoms that is important to improve, presenting the effect of the intervention at an individual level and can provide the patients and therapists to provide more direct information to decide whether to use the treatment or not. The MCII of the NRS is set at 2 points, and the MCII of the WOMAC functional subscale (Likert version 3.1) is set at 6 (33). Cun-Zhi Liu’s team used the response rate as the main evaluation index in an article published in the journal Arthritis & Rheumatology on the treatment of osteoarthritis of the knee with acupuncture (19). At present, many domestic and foreign scholars use response rate as the main observation index of KOA (32, 34). Secondary outcome indicators include the NRS, a modification of the VAS by Budzynski et al. The NRS is more responsive than the VAS and VRS (35). The WOMAC score was developed in 1988 by Bellamy et al. (36). It has a high degree of validity and reliability, which reflects treatment outcomes well and the WOMAC score is suitable for the assessment of chronic injuries (37). The 6MWT not only shows an individual’s level of endurance, but also assesses a patient’s walking ability (38). The patient is asked to walk for 6 minutes down a 30-meter corridor, and the distance he or she walks in the 6 minutes is measured (39). The 6MWT is currently recommended by the Osteoarthritis Research Society International (OARSI) (40) as one of a set of performance-based tests to assess a patient’s walking ability. In addition, pain and dysfunction will affect the quality of life of patients, and today’s society has higher and higher requirements for quality of life, so the assessment of the quality of life of patients before and after treatment is also crucial. In this study, we chose the SF-12 scale to assess the quality of life of patients, which is simpler and easier to use than the SF-36, and saves the time and cost and improves the rate of completion of the scale, which has been more widely used in the clinic (41). In this study, we will assess the patients’ situation from three aspects: pain, function and quality of life, which can respond to the patients’ problems more comprehensively.

There are some limitations to this study. Firstly, it was not possible to blind both the operator and the participants. Therefore, the applicability of the results to other populations needs to be carefully considered. Secondly, patients recruited from various centers may differ in gender, age, and other disorders. Therefore, subgroup analyses may be needed to determine the effectiveness of FSN for different subgroups. Thirdly, this study did not have a sham-Fu’s subcutaneous-needle group and therefore could not remove the placebo effect. Considering these limitations, we will carefully interpret our results.
