# Supplementary material for: Fu’s subcutaneous needling approach versus electroacupuncture for knee osteoarthritis: protocol for comparative effectiveness and safety randomized controlled trial
Source: Front Med (Lausanne). 2025 May 29;12:1510326. doi: 10.3389/fmed.2025.1510326 (PMC12159060; doi:10.3389/fmed.2025.1510326)
Supplement: Supplementary file 1 [file Data_Sheet_1.docx]

**A Multicenter Study of Fu's subcutaneous needling in the Treatment of Knee Osteoarthritis**

**Case Report Form**

**version number：1.2，version date：20231228**

| □01Shandong Provincial Third Hospital, Shandong University |
| --- |
| □02Guangdong Hospital of Traditional Chinese Medicine |
| □03Nanjing Hospital of Chinese Medcine affiliated to Nanjing University of Chinese Medcine |
| □04The Second Affiliated Hospital of Bengbu Medical College |
| □05The First People's Hospital of Wenling Bachelor's degree |
| □06Tai'an Hospital of Traditional Chinese Medicine |

**Case Random Number：**|___|____|____|___|

**Subject's initials：**|___|____|____|___|

**Physician's name (block letters)：______________**

**groups：FSN** □  **EA** □

**Study period: January 2024 ----- June 2026**

**Instructions for completing the case report form**

**(Please read the following instructions for completing the form carefully before completing it formally)**

1. This case report form must be completed by a full-time evaluator and, whenever possible, by the same person; the physician's signature on the cover and end pages is to be completed by the investigator responsible for the case.

2. The subject enrollment number in the upper right corner of the cover page is prepared by the investigator at each center to facilitate monitoring and CRF management at the center.

3. This form should be filled out with a pen or marker, not with a pencil or ballpoint pen.

4. Those who have been screened formally complete the case report form, and those who have discontinued treatment carry forward the last data as their last data, and truthfully record the time and reason for withdrawal from the study.

5. Wherever there is a “□” option in the table, please put a “×” in the correct option “□”.

6. Please fill in the numbers in the open boxes, one number in each box, and if the number of digits is not enough, please add zeros to the preceding or following boxes, e.g.: weight 58 kg, fill in the blanks as: weight: | 0 | 5 | 8 |. | 0 | kg.

7. All checking items must be filled in, please fill in “ND” for not done/not checked/missed for any reason, “UK” for unknown/unknown data, and “NA” if the option is not applicable. If the option is not applicable, please fill in “NA”.

8. How to fill in the subject's name: The four cells of the pinyin abbreviation of the subject's name should be filled in. For two-character names, fill in the first two letters of the pinyin of each character; for three-character names, fill in the first letter of the pinyin of each character and the second letter of the pinyin of the third character; for four-character names, fill in the first letter of their respective pinyin; and for names of more than four-character names, fill in the first letter of the pinyin of the first four characters. Examples: Zhang Fang, Li Zhengfen, Shangguan Xiaoyun. The investigator must fill in the subject identity registration form to facilitate identification of subjects during and after the trial.

9. Fill in must be accurate, clear, not arbitrarily erased or altered, errors need to be corrected with a horizontal line centered, and sign the name of the modifier (physician's initials, capital letters) and the time of modification, if necessary, to explain the reasons. Do not cover up the original data, prohibit the use of erasers, correction fluid or scratch to remove the original content.

Example: Filling in errors 2011/07/13[Year, month, day]

Correction mode2011/07/~~13~~ ^14 SJF 2011/07/14^

The carbonless copy front page will be centrally managed by the lead unit at the end of the study and center researchers may not make any entries or changes on the case report form retained by the research unit, and any entries or changes must be made using the Data Clarification Form (DCF).

10. The date adopts the international ISO 8601 date format: yyyymmdd. e.g. October 15, 2010 is written as: 20101015.

Please fill in “UK” in the unknown date section, e.g. 201010UK, 2010UKUK, UKUKUK. For example: 201010UK, 2010UKUK, UKUKUK. Informed consent, medication history, concomitant medications, adverse events, etc. must be fully dated. The time should be in 24-hour format, e.g. 21:00 instead of 09:00pm, and 00:00 (instead of 24:00) should be recorded as the new day, e.g. 00:00 on September 2, 2012 instead of 24:00 on September 1, 2012.

11. Do not use cross-referenced terms, e.g., as before, as above, etc. Please use medical terminology when describing medical history, adverse events

Please use medical terminology when describing medical history, adverse events, diagnoses and drug names; use standardized full names, no abbreviations or acronyms unless they are listed in the study protocol.

12. Please strictly execute the clinical trial protocol. For the items to be completed at each visit, please execute them against the clinical study flow chart and pay attention to observe the time window.

13. During the clinical study, please faithfully fill in the combined medication record form and the adverse reaction/event record form. If serious adverse events occur, please fill in the Serious Adverse Event Record Form while treating them as usual, regardless of whether they are related to the study treatment or not.

**Clinical Trial Flowchart**

| Period | **Screening Periods** | **Treatment period** | | | | **Follow-up period** |
| --- | --- | --- | --- | --- | --- | --- |
| **Time (weeks)** | Week 0 | Week 1 | Week 2 | Week 3 | Week 4 | Week 8 |
|  | **Basic information collection** | | | | | |
| Inclusion/exclusion criteria | × |  |  |  |  |  |
| Signed informed consent | × |  |  |  |  |  |
| Demographicinformation | × |  |  |  |  |  |
| Medical and therapeutic history | × |  |  |  |  |  |
| Knee X-ray | × |  |  |  |  |  |
|  | **Security Observations** | | | | |  |
| Adverse event |  | × | × | × | × |  |
|  | **Therapeutic Observations** | | | | |  |
| Painful walking on level ground (NRS) | × | × | × | × | × | × |
| Pain going up or down stairs (NRS) | × | × | × | × | × | × |
| 6-minute walk test | × | × | × | × | × | × |
| WOMAC score | × | × | × | × | × | × |
| SF-12 score | × |  |  |  | × | × |
|  | **else** | | | | |  |

**Date of signing informed consent：**202|__| year |___|___| month |___|___| day

| **Inclusion Criteria (a “no” to any of the criteria precludes inclusion in the study)** | **Yes** | **No** |
| --- | --- | --- |
| 1. Meets diagnostic criteria; | □**_1_** | □**_0_** |
| 1. Age≤ 75 years old, regardless of gender; | □**_1_** | □**_0_** |
| 1. Mean knee pain level NRS score ≥4 before enrollment; | □**_1_** | □**_0_** |
| 1. Classification of the condition, KOA imaging classification (Kellgren Lawrence, KL) grade 2 to 3; | □**_1_** | □**_0_** |
| 1. Have not received acupuncture, herbal medicine, or other treatments in the last 1 month; | □**_1_** | □**_0_** |
| 1. Agree to participate in this study and sign the informed consent form. |  |  |

| **Exclusion Criteria (a “yes” to any of the criteria precludes inclusion in the study)** | **No** | **Yes** |
| --- | --- | --- |
| 1. Combined with serious cardiovascular, cerebrovascular, digestive, respiratory, urinary, hematopoietic system and other diseases; | □**_0_** | □**_1_** |
| 2. Knee pain caused by other diseases such as gouty arthritis, rheumatoid arthritis, and knee deformity; | □**_0_** | □**_1_** |
| 3. History of surgery, joint cavity injection, or severe trauma to the target knee within the last 1 year; | □**_0_** | □**_1_** |
| 4. Presence of contraindications to MRI examination, e.g., pacemakers, metal implants, fear of confined spaces, pregnancy preparation, pregnancy, etc; | □**_0_** | □**_1_** |
| 5. Those who refuse to sign the informed consent form; | □_0_ | □_1_ |

| **Demographic Information** | | | | | | |
| --- | --- | --- | --- | --- | --- | --- |
| **Gender** | □**_1_**man□**_2_** woman | **date of birth** |  | | | |
| **Ethnicity** | □**_1_**Han ethnic group□**_2_**Manchu ethnic group□**_3_**Zhuang ethnic group□**_4_**Hui ethnic group□**_5_**Hmong or Miao ethnic group□**_6_**Uighur ethnic group□**_7_**else： | | | | | |
| **Marriage** | □**_1_** Yes□**_0_** No | | | | | |
| **Education** | □**_1_** Graduate student and above□**_2_** Bachelor's degree□**_3_**University junior college□**_4_** High school/middle school/technical school□**_5_**unior high school  □**_6_** Elementary school□**_7_** lliterate | | | | | |
| **Occupation** | □**_1_**Worker□**_2_**Farmer□**_3_**Teacher□**_4_**Soldier□**_5_**Student□**_6_**Cadre  □**_7_**Medical worker□**_8_**Lawyer□**_9_**Employee□**_10_**Retired□**_11_** Freelancer  □**_12_**else | | | | | |
| **Weight** | \|___\|____\|___\|.\|__\|kg | **Height** | | \|__\|_._\|___\|___\|m | **BMI** |  |

5

| **medical history** | | | | | | | |
| --- | --- | --- | --- | --- | --- | --- | --- |
| **disease duration** | \|___\|___\|year\|___\|___\|month (Time from 1st episode to current visit) | | | | | | |
| **typical symptom** | □**_1_**Knee pain□2 Morning stiffness□3 Bone friction sounds□4 Limited mobility□5 Floating patella test (+) (multiple choice) | | | | | | |
| **patella pushing test** | □_1_Outer upper□2 Outer lower□3 Inner upper□4 Inner lower (multiple choice) | | | | | | |
| **Pain Location** | □_1_Outer upper□2 Outer lower□3 Inner upper□4 Inner lower (multiple choice) | | | | | | |
| **co-morbidities** | □**_0_** No□**_1_**Yes，Please continue with the form below | | | **Worst symptomatic side** | | | □**_0_**Left knee□**_1_**Right knee |
| **co-morbidities name** | **Start time** | | **Is it continuous** | | **end time** | | |
|  |  | | □Yes□No | |  | | |
|  |  | | □Yes□No | |  | | |
|  |  | | □Yes□No | |  | | |
|  |  | | □Yes□No | |  | | |
|  |  | | □Yes□No | |  | | |
|  |  | | □Yes□No | |  | | |
|  |  | | □Yes□No | |  | | |
| Treatment history (related to osteoarthritis of the knee) | | | | | | | |
| Treatment related to osteoarthritis of the knee that was applied by the subject within 1 month prior to enrollment but discontinued at the time of enrollment  □**_0_** No □**_1_**Yes, Please continue with the form below | | | | | | | |
| Treatment code | Name of drug  (for codes 1, 2 and 4 only) | Starting time | | | | Deactivation time | |
| \|___\|___\| |  |  | | | |  | |
| \|___\|___\| |  |  | | | |  | |
| \|___\|___\| |  |  | | | |  | |
| \|___\|___\| |  |  | | | |  | |
| \|___\|___\| |  |  | | | |  | |
| **Treatment Code:**□**_1_** Oral treatment with western medicine□_2_ Articular injection□_3_ Surgical treatment□_4_ Proprietary Chinese medicine□_5_ Traditional Chinese medicine  □_6_Acupuncture□_7_Acupuncture□_8_Massage□_9_Transcutaneous Electrical Nerve Stimulation□_10_Heat Therapy□_11_Hydrotherapy□_12_Traction□_13_Ultrasound□_14_Other, please describe | | | | | | | |

| **radiographic examination** |
| --- |
| Frontal and lateral X-rays of the knee joint **Check date:** □**_0_** Left knee□**_1_**Right knee |
| □**_1_** Normal□_2_ Abnormal, not clinically significant□_3_ Abnormal, clinically significant, please describe: |

**Baseline/before first treatment**

7

| **Indicator of efficacy evaluation (bilateral pain, only the most symptomatic side is recorded)** □**_0_** Left knee □**_1_**Right knee | | | | | | |
| --- | --- | --- | --- | --- | --- | --- |
| 1. walking on level ground pain NRS score: | | \|___\|___\|score | | | | |
| 2. up and down stairs pain NRS score: | | go up the stairs：\|___\|___\|score  go down the stairs：\|___\|___\|score | | | | |
| 3. 6-minute walk test | | \|___\|____\|___\|.\|__\|meters | | | | |
| **WOMACO steoarthritis Index** | | | | | | |
| **item (of a dictionary, encyclopedia etc)** | **No** | **Trivial** | **Medium degree** | **Visibly** | **Extremely serious** | |
| **Pain** | **0** | **1** | **2** | **3** | **4** | |
| 1. Walking on a flat surface | □ | □ | □ | □ | □ | |
| 2. Going up or down stairs | □ | □ | □ | □ | □ | |
| 3. Sleeping at night | □ | □ | □ | □ | □ | |
| 4. Sitting or lying down | □ | □ | □ | □ | □ | |
| 5. Standing up straight | □ | □ | □ | □ | □ | |
| **Stiffness** | **0** | **1** | **2** | **3** | **4** | |
| 6. When you first wake up in the morning | □ | □ | □ | □ | □ | |
| 7. When sitting, lying down or resting | □ | □ | □ | □ | □ | |
| **Difficulty in performing daily activities** | **0** | **1** | **2** | **3** | **4** | |
| 8. Going down the stairs | □ | □ | □ | □ | □ | |
| 9.Going up the stairs | □ | □ | □ | □ | □ | |
| 10.From sitting to standing (getting up) | □ | □ | □ | □ | □ | |
| 11.Standing | □ | □ | □ | □ | □ | |
| 12.Bending to the ground | □ | □ | □ | □ | □ | |
| 13.Walking on a flat surface | □ | □ | □ | □ | □ | |
| 14. Getting in and out of a car, or on and off a bus | □ | □ | □ | □ | □ | |
| 15. Going out shopping | □ | □ | □ | □ | □ | |
| 16. Putting on socks | □ | □ | □ | □ | □ | |
| 17. Getting up from bed | □ | □ | □ | □ | □ | |
| 18. Take off your socks | □ | □ | □ | □ | □ | |
| 19. Lying on the bed | □ | □ | □ | □ | □ | |
| 20.Taking a bath | □ | □ | □ | □ | □ | |
| 21.Sitting | □ | □ | □ | □ | □ | |
| 22. Squatting or getting up in the bathroom | □ | □ | □ | □ | □ | |
| 23. Doing heavy chores | □ | □ | □ | □ | □ | |
| 24. Doing easy chores | □ | □ | □ | □ | □ | |
| **Totals** | \|___\|___\|score | | | | | |
| **SF-12 Quality of Life Score**  Instructions: The following questions ask for your opinion about your health. Please answer each question below and select the best answer if you are not sure of choosing the exact answer. | | | | | | |
| **Content of the assessment** | Options | | | | | Score |
| 1. In general, you consider your current health condition to be: | Perfectly good□ (1 point) Very good□ (2 points)  Good□ (3 points) Average□ (4 points) Poor□ (5 points) | | | | |  |
| 2. Each of the following is an activity that you may do in your daily life. Are there any limitations to performing the following activities in terms of your current health status? If yes, to what extent? | | | | | | |
| 2a.Moderate-intensity activities such as lifting a table, moving a vacuum cleaner, bowling or golfing | A lot of restrictions□ (1 point)  A little bit of restrictions□ (2 points)  No restriction at all□ (3 points) | | | | |  |
| 2b.Climb a few flights of stairs. | A lot of restrictions□ (1 point)  A little bit of restrictions□ (2 points)  No restriction at all□ (3 points) | | | | |  |
| 3.In the past 4 weeks, do any of the following problems occur at work or other regular daily activities due to a health condition? | | | | | | |
| 3a.Getting less done in work or life than you want to get done | Yes□（1 point） No□（2 points） | | | | |  |
| 3b.Restrictions on completing work or daily activities | Yes□（1 point） No□（2 points） | | | | |  |
| 4.In the past 4 weeks, due to emotional reasons (e.g., feeling depressed/anxious), do any of the following problems occur at work or in daily life? | | | | | | |
| 4a.Things get done less than expected | Yes□（1 point） No□（2 points） | | | | |  |
| 4b.Completing work or daily activities less carefully than usual | Yes□（1 point） No□（2 points） | | | | |  |
| 5. How has the pain affected your normal work (both at work and at home) in the past 4 weeks? | Not at all□ (1 point)□ (2 points)□ (2 points)□ (2 points)□ (3 points)□ (3 points)□ (3 points)□ (3 points)□ (3 points)□ (3 points)□ (3 points)□ (3 points) | | | | |  |
| 6. The following questions are about how you have been feeling and how things have been going in the last 4 weeks. For each question, please choose the answer that comes closest to how you have been feeling. | | | | | | |
| 6a.Feeling at peace? | All the time□ (1 point)  Most of the time□ (2 points)  A lot of time□ (3 points)  Some of the time□ (4 points)  Rarely (5 points)  Never (6 points) | | | | |  |
| 6b.Feeling energized? | All the time□ (1 point)  Most of the time□ (2 points)  A lot of time□ (3 points)  Some of the time□ (4 points)  Rarely (5 points)  Never (6 points) | | | | |  |
| 6c.Feeling down and out? | All the time□ (1 point)  Most of the time□ (2 points)  A lot of time□ (3 points)  Some of the time□ (4 points)  Rarely (5 points)  Never (6 points) | | | | |  |
| 7. In the past 4 weeks, how much of the time did your physical health condition or emotional problems prevent you from socializing (e.g., visiting friends, family, etc.)? | All the time□ (1 point)  Most of the time□ (2 points)  A lot of time□ (3 points)  Some of the time□ (4 points)  Rarely (5 points)  Never (6 points) | | | | |  |

**Tip: If comorbidities and/or adverse events occurred between the time of signing the informed consent and enrollment, please fill in the relevant page numbers!**

**Date of visit:**

*Note: This date should be the date of randomization (i.e., the date of enrollment) after the subject was screened and qualified, and all other visit time windows should be extrapolated from this date.

| **Week 1 follow-up (bilateral pain, only the most symptomatic side was recorded)**  □**_0_** Left knee □**_1_**Right knee | | | | | |
| --- | --- | --- | --- | --- | --- |
| **Indicators for evaluating therapeutic efficacy** | | | | | |
| 1. walking on level ground pain NRS score: | | \|___\|___\|score | | | |
| 2. up and down stairs pain NRS score: | | go up the stairs：\|___\|___\|score  go down the stairs：\|___\|___\|score | | | |
| 3. 6-minute walk test | | \|___\|____\|___\|.\|__\|meters | | | |
| **WOMACO steoarthritis Index** | | | | | |
| **item (of a dictionary, encyclopedia etc)** | **No** | **Trivial** | **Medium degree** | **Visibly** | **Extremely serious** |
| **Pain** | **0** | **1** | **2** | **3** | **4** |
| 1. Walking on a flat surface | □ | □ | □ | □ | □ |
| 2. Going up or down stairs | □ | □ | □ | □ | □ |
| 3. Sleeping at night | □ | □ | □ | □ | □ |
| 4. Sitting or lying down | □ | □ | □ | □ | □ |
| 5. Standing up straight | □ | □ | □ | □ | □ |
| **Stiffness** | **0** | **1** | **2** | **3** | **4** |
| 6. When you first wake up in the morning | □ | □ | □ | □ | □ |
| 7. When sitting, lying down or resting | □ | □ | □ | □ | □ |
| **Difficulty in performing daily activities** | **0** | **1** | **2** | **3** | **4** |
| 8. Going down the stairs | □ | □ | □ | □ | □ |
| 9.Going up the stairs | □ | □ | □ | □ | □ |
| 10.From sitting to standing (getting up) | □ | □ | □ | □ | □ |
| 11.Standing | □ | □ | □ | □ | □ |
| 12.Bending to the ground | □ | □ | □ | □ | □ |
| 13.Walking on a flat surface | □ | □ | □ | □ | □ |
| 14. Getting in and out of a car, or on and off a bus | □ | □ | □ | □ | □ |
| 15. Going out shopping | □ | □ | □ | □ | □ |
| 16. Putting on socks | □ | □ | □ | □ | □ |
| 17. Getting up from bed | □ | □ | □ | □ | □ |
| 18. Take off your socks | □ | □ | □ | □ | □ |
| 19. Lying on the bed | □ | □ | □ | □ | □ |
| 20.Taking a bath | □ | □ | □ | □ | □ |
| 21.Sitting | □ | □ | □ | □ | □ |
| 22. Squatting or getting up in the bathroom | □ | □ | □ | □ | □ |
| 23. Doing heavy chores | □ | □ | □ | □ | □ |
| 24. Doing easy chores | □ | □ | □ | □ | □ |
| **Totals** | \|___\|___\|score | | | | |

**Tip: If comorbidities and/or adverse events occurred between the time of signing the informed consent and enrollment, please fill in the relevant page numbers!**

**Date of visit:**

| **Week 2 follow-up (bilateral pain, only the most symptomatic side was recorded)**  □**_0_** Left knee □**_1_**Right knee | | | | | |
| --- | --- | --- | --- | --- | --- |
| **Indicators for evaluating therapeutic efficacy** | | | | | |
| 1. walking on level ground pain NRS score: | | \|___\|___\|score | | | |
| 2. up and down stairs pain NRS score: | | go up the stairs：\|___\|___\|score  go down the stairs：\|___\|___\|score | | | |
| 3. 6-minute walk test | | \|___\|____\|___\|.\|__\|meters | | | |
| **WOMACO steoarthritis Index** | | | | | |
| **item (of a dictionary, encyclopedia etc)** | **No** | **Trivial** | **Medium degree** | **Visibly** | **Extremely serious** |
| **Pain** | **0** | **1** | **2** | **3** | **4** |
| 1. Walking on a flat surface | □ | □ | □ | □ | □ |
| 2. Going up or down stairs | □ | □ | □ | □ | □ |
| 3. Sleeping at night | □ | □ | □ | □ | □ |
| 4. Sitting or lying down | □ | □ | □ | □ | □ |
| 5. Standing up straight | □ | □ | □ | □ | □ |
| **Stiffness** | **0** | **1** | **2** | **3** | **4** |
| 6. When you first wake up in the morning | □ | □ | □ | □ | □ |
| 7. When sitting, lying down or resting | □ | □ | □ | □ | □ |
| **Difficulty in performing daily activities** | **0** | **1** | **2** | **3** | **4** |
| 8. Going down the stairs | □ | □ | □ | □ | □ |
| 9.Going up the stairs | □ | □ | □ | □ | □ |
| 10.From sitting to standing (getting up) | □ | □ | □ | □ | □ |
| 11.Standing | □ | □ | □ | □ | □ |
| 12.Bending to the ground | □ | □ | □ | □ | □ |
| 13.Walking on a flat surface | □ | □ | □ | □ | □ |
| 14. Getting in and out of a car, or on and off a bus | □ | □ | □ | □ | □ |
| 15. Going out shopping | □ | □ | □ | □ | □ |
| 16. Putting on socks | □ | □ | □ | □ | □ |
| 17. Getting up from bed | □ | □ | □ | □ | □ |
| 18. Take off your socks | □ | □ | □ | □ | □ |
| 19. Lying on the bed | □ | □ | □ | □ | □ |
| 20.Taking a bath | □ | □ | □ | □ | □ |
| 21.Sitting | □ | □ | □ | □ | □ |
| 22. Squatting or getting up in the bathroom | □ | □ | □ | □ | □ |
| 23. Doing heavy chores | □ | □ | □ | □ | □ |
| 24. Doing easy chores | □ | □ | □ | □ | □ |
| **Totals** | \|___\|___\|score | | | | |

**Tip: If comorbidities and/or adverse events occurred between the time of signing the informed consent and enrollment, please fill in the relevant page numbers!**

**Date of visit:**

| **Week 3 follow-up (bilateral pain, only the most symptomatic side was recorded)**  □**_0_** Left knee □**_1_**Right knee | | | | | |
| --- | --- | --- | --- | --- | --- |
| **Indicators for evaluating therapeutic efficacy** | | | | | |
| 1. walking on level ground pain NRS score: | | \|___\|___\|score | | | |
| 2. up and down stairs pain NRS score: | | go up the stairs：\|___\|___\|score  go down the stairs：\|___\|___\|score | | | |
| 3. 6-minute walk test | | \|___\|____\|___\|.\|__\|meters | | | |
| **WOMACO steoarthritis Index** | | | | | |
| **item (of a dictionary, encyclopedia etc)** | **No** | **Trivial** | **Medium degree** | **Visibly** | **Extremely serious** |
| **Pain** | **0** | **1** | **2** | **3** | **4** |
| 1. Walking on a flat surface | □ | □ | □ | □ | □ |
| 2. Going up or down stairs | □ | □ | □ | □ | □ |
| 3. Sleeping at night | □ | □ | □ | □ | □ |
| 4. Sitting or lying down | □ | □ | □ | □ | □ |
| 5. Standing up straight | □ | □ | □ | □ | □ |
| **Stiffness** | **0** | **1** | **2** | **3** | **4** |
| 6. When you first wake up in the morning | □ | □ | □ | □ | □ |
| 7. When sitting, lying down or resting | □ | □ | □ | □ | □ |
| **Difficulty in performing daily activities** | **0** | **1** | **2** | **3** | **4** |
| 8. Going down the stairs | □ | □ | □ | □ | □ |
| 9.Going up the stairs | □ | □ | □ | □ | □ |
| 10.From sitting to standing (getting up) | □ | □ | □ | □ | □ |
| 11.Standing | □ | □ | □ | □ | □ |
| 12.Bending to the ground | □ | □ | □ | □ | □ |
| 13.Walking on a flat surface | □ | □ | □ | □ | □ |
| 14. Getting in and out of a car, or on and off a bus | □ | □ | □ | □ | □ |
| 15. Going out shopping | □ | □ | □ | □ | □ |
| 16. Putting on socks | □ | □ | □ | □ | □ |
| 17. Getting up from bed | □ | □ | □ | □ | □ |
| 18. Take off your socks | □ | □ | □ | □ | □ |
| 19. Lying on the bed | □ | □ | □ | □ | □ |
| 20.Taking a bath | □ | □ | □ | □ | □ |
| 21.Sitting | □ | □ | □ | □ | □ |
| 22. Squatting or getting up in the bathroom | □ | □ | □ | □ | □ |
| 23. Doing heavy chores | □ | □ | □ | □ | □ |
| 24. Doing easy chores | □ | □ | □ | □ | □ |
| **Totals** | \|___\|___\|score | | | | |

**Tip: If comorbidities and/or adverse events occurred between the time of signing the informed consent and enrollment, please fill in the relevant page numbers!**

**Date of visit:**

| **Week 4 follow-up (bilateral pain, only the most symptomatic side was recorded)**  □**_0_** Left knee □**_1_**Right knee | | | | | | |
| --- | --- | --- | --- | --- | --- | --- |
| **Indicators for evaluating therapeutic efficacy** | | | | | | |
| 1. walking on level ground pain NRS score: | | \|___\|___\|score | | | | |
| 2. up and down stairs pain NRS score: | | go up the stairs：\|___\|___\|score  go down the stairs：\|___\|___\|score | | | | |
| 3. 6-minute walk test | | \|___\|____\|___\|.\|__\|meters | | | | |
| **WOMACO steoarthritis Index** | | | | | | |
| **item (of a dictionary, encyclopedia etc)** | **No** | **Trivial** | **Medium degree** | **Visibly** | **Extremely serious** | |
| **Pain** | **0** | **1** | **2** | **3** | **4** | |
| 1. Walking on a flat surface | □ | □ | □ | □ | □ | |
| 2. Going up or down stairs | □ | □ | □ | □ | □ | |
| 3. Sleeping at night | □ | □ | □ | □ | □ | |
| 4. Sitting or lying down | □ | □ | □ | □ | □ | |
| 5. Standing up straight | □ | □ | □ | □ | □ | |
| **Stiffness** | **0** | **1** | **2** | **3** | **4** | |
| 6. When you first wake up in the morning | □ | □ | □ | □ | □ | |
| 7. When sitting, lying down or resting | □ | □ | □ | □ | □ | |
| **Difficulty in performing daily activities** | **0** | **1** | **2** | **3** | **4** | |
| 8. Going down the stairs | □ | □ | □ | □ | □ | |
| 9.Going up the stairs | □ | □ | □ | □ | □ | |
| 10.From sitting to standing (getting up) | □ | □ | □ | □ | □ | |
| 11.Standing | □ | □ | □ | □ | □ | |
| 12.Bending to the ground | □ | □ | □ | □ | □ | |
| 13.Walking on a flat surface | □ | □ | □ | □ | □ | |
| 14. Getting in and out of a car, or on and off a bus | □ | □ | □ | □ | □ | |
| 15. Going out shopping | □ | □ | □ | □ | □ | |
| 16. Putting on socks | □ | □ | □ | □ | □ | |
| 17. Getting up from bed | □ | □ | □ | □ | □ | |
| 18. Take off your socks | □ | □ | □ | □ | □ | |
| 19. Lying on the bed | □ | □ | □ | □ | □ | |
| 20.Taking a bath | □ | □ | □ | □ | □ | |
| 21.Sitting | □ | □ | □ | □ | □ | |
| 22. Squatting or getting up in the bathroom | □ | □ | □ | □ | □ | |
| 23. Doing heavy chores | □ | □ | □ | □ | □ | |
| 24. Doing easy chores | □ | □ | □ | □ | □ | |
| **Totals** | \|___\|___\|score | | | | | |
| **SF-12 Quality of Life Score**  Instructions: The following questions ask for your opinion about your health. Please answer each question below and select the best answer if you are not sure of choosing the exact answer. | | | | | | |
| **Content of the assessment** | Options | | | | |  |
| 1. In general, you consider your current health condition to be: | Perfectly good□ (1 point) Very good□ (2 points)  Good□ (3 points) Average□ (4 points) Poor□ (5 points) | | | | |  |
| 2. Each of the following is an activity that you may do in your daily life. Are there any limitations to performing the following activities in terms of your current health status? If yes, to what extent? | | | | | | |
| 2a.Moderate-intensity activities such as lifting a table, moving a vacuum cleaner, bowling or golfing | A lot of restrictions□ (1 point)  A little bit of restrictions□ (2 points)  No restriction at all□ (3 points) | | | | |  |
| 2b.Climb a few flights of stairs. | A lot of restrictions□ (1 point)  A little bit of restrictions□ (2 points)  No restriction at all□ (3 points) | | | | |  |
| 3.In the past 4 weeks, do any of the following problems occur at work or other regular daily activities due to a health condition? | | | | | | |
| 3a.Getting less done in work or life than you want to get done | Yes□（1 point） No□（2 points） | | | | |  |
| 3b.Restrictions on completing work or daily activities | Yes□（1 point） No□（2 points） | | | | |  |
| 4.In the past 4 weeks, due to emotional reasons (e.g., feeling depressed/anxious), do any of the following problems occur at work or in daily life? | | | | | | |
| 4a.Things get done less than expected | Yes□（1 point） No□（2 points） | | | | |  |
| 4b.Completing work or daily activities less carefully than usual | Yes□（1 point） No□（2 points） | | | | |  |
| 5. How has the pain affected your normal work (both at work and at home) in the past 4 weeks? | Not at all□ (1 point)□ (2 points)□ (2 points)□ (2 points)□ (3 points)□ (3 points)□ (3 points)□ (3 points)□ (3 points)□ (3 points)□ (3 points)□ (3 points) | | | | |  |
| 6. The following questions are about how you have been feeling and how things have been going in the last 4 weeks. For each question, please choose the answer that comes closest to how you have been feeling. | | | | | | |
| 6a.Feeling at peace? | All the time□ (1 point)  Most of the time□ (2 points)  A lot of time□ (3 points)  Some of the time□ (4 points)  Rarely (5 points)  Never (6 points) | | | | |  |
| 6b.Feeling energized? | All the time□ (1 point)  Most of the time□ (2 points)  A lot of time□ (3 points)  Some of the time□ (4 points)  Rarely (5 points)  Never (6 points) | | | | |  |
| 6c.Feeling down and out? | All the time□ (1 point)  Most of the time□ (2 points)  A lot of time□ (3 points)  Some of the time□ (4 points)  Rarely (5 points)  Never (6 points) | | | | |  |
| 7. In the past 4 weeks, how much of the time did your physical health condition or emotional problems prevent you from socializing (e.g., visiting friends, family, etc.)? | All the time□ (1 point)  Most of the time□ (2 points)  A lot of time□ (3 points)  Some of the time□ (4 points)  Rarely (5 points)  Never (6 points) | | | | |  |

**Tip: If comorbidities and/or adverse events occurred between the time of signing the informed consent and enrollment, please fill in the relevant page numbers!**

**Date of visit:**

18

| **Week 8 follow-up (bilateral pain, only the most symptomatic side was recorded)**  □**_0_** Left knee □**_1_**Right knee | | | | | | |
| --- | --- | --- | --- | --- | --- | --- |
| **Indicators for evaluating therapeutic efficacy** | | | | | | |
| 1. walking on level ground pain NRS score: | | \|___\|___\|score | | | | |
| 2. up and down stairs pain NRS score: | | go up the stairs：\|___\|___\|score  go down the stairs：\|___\|___\|score | | | | |
| 3. 6-minute walk test | | \|___\|____\|___\|.\|__\|meters | | | | |
| **WOMACO steoarthritis Index** | | | | | | |
| **item (of a dictionary, encyclopedia etc)** | **No** | **Trivial** | **Medium degree** | **Visibly** | **Extremely serious** | |
| **Pain** | **0** | **1** | **2** | **3** | **4** | |
| 1. Walking on a flat surface | □ | □ | □ | □ | □ | |
| 2. Going up or down stairs | □ | □ | □ | □ | □ | |
| 3. Sleeping at night | □ | □ | □ | □ | □ | |
| 4. Sitting or lying down | □ | □ | □ | □ | □ | |
| 5. Standing up straight | □ | □ | □ | □ | □ | |
| **Stiffness** | **0** | **1** | **2** | **3** | **4** | |
| 6. When you first wake up in the morning | □ | □ | □ | □ | □ | |
| 7. When sitting, lying down or resting | □ | □ | □ | □ | □ | |
| **Difficulty in performing daily activities** | **0** | **1** | **2** | **3** | **4** | |
| 8. Going down the stairs | □ | □ | □ | □ | □ | |
| 9.Going up the stairs | □ | □ | □ | □ | □ | |
| 10.From sitting to standing (getting up) | □ | □ | □ | □ | □ | |
| 11.Standing | □ | □ | □ | □ | □ | |
| 12.Bending to the ground | □ | □ | □ | □ | □ | |
| 13.Walking on a flat surface | □ | □ | □ | □ | □ | |
| 14. Getting in and out of a car, or on and off a bus | □ | □ | □ | □ | □ | |
| 15. Going out shopping | □ | □ | □ | □ | □ | |
| 16. Putting on socks | □ | □ | □ | □ | □ | |
| 17. Getting up from bed | □ | □ | □ | □ | □ | |
| 18. Take off your socks | □ | □ | □ | □ | □ | |
| 19. Lying on the bed | □ | □ | □ | □ | □ | |
| 20.Taking a bath | □ | □ | □ | □ | □ | |
| 21.Sitting | □ | □ | □ | □ | □ | |
| 22. Squatting or getting up in the bathroom | □ | □ | □ | □ | □ | |
| 23. Doing heavy chores | □ | □ | □ | □ | □ | |
| 24. Doing easy chores | □ | □ | □ | □ | □ | |
| **Totals** | \|___\|___\|score | | | | | |
| **SF-12 Quality of Life Score**  Instructions: The following questions ask for your opinion about your health. Please answer each question below and select the best answer if you are not sure of choosing the exact answer. | | | | | | |
| **Content of the assessment** | Options | | | | |  |
| 1. In general, you consider your current health condition to be: | Perfectly good□ (1 point) Very good□ (2 points)  Good□ (3 points) Average□ (4 points) Poor□ (5 points) | | | | |  |
| 2. Each of the following is an activity that you may do in your daily life. Are there any limitations to performing the following activities in terms of your current health status? If yes, to what extent? | | | | | | |
| 2a.Moderate-intensity activities such as lifting a table, moving a vacuum cleaner, bowling or golfing | A lot of restrictions□ (1 point)  A little bit of restrictions□ (2 points)  No restriction at all□ (3 points) | | | | |  |
| 2b.Climb a few flights of stairs. | A lot of restrictions□ (1 point)  A little bit of restrictions□ (2 points)  No restriction at all□ (3 points) | | | | |  |
| 3.In the past 4 weeks, do any of the following problems occur at work or other regular daily activities due to a health condition? | | | | | | |
| 3a.Getting less done in work or life than you want to get done | Yes□（1 point） No□（2 points） | | | | |  |
| 3b.Restrictions on completing work or daily activities | Yes□（1 point） No□（2 points） | | | | |  |
| 4.In the past 4 weeks, due to emotional reasons (e.g., feeling depressed/anxious), do any of the following problems occur at work or in daily life? | | | | | | |
| 4a.Things get done less than expected | Yes□（1 point） No□（2 points） | | | | |  |
| 4b.Completing work or daily activities less carefully than usual | Yes□（1 point） No□（2 points） | | | | |  |
| 5. How has the pain affected your normal work (both at work and at home) in the past 4 weeks? | Not at all□ (1 point)□ (2 points)□ (2 points)□ (2 points)□ (3 points)□ (3 points)□ (3 points)□ (3 points)□ (3 points)□ (3 points)□ (3 points)□ (3 points) | | | | |  |
| 6. The following questions are about how you have been feeling and how things have been going in the last 4 weeks. For each question, please choose the answer that comes closest to how you have been feeling. | | | | | | |
| 6a.Feeling at peace? | All the time□ (1 point)  Most of the time□ (2 points)  A lot of time□ (3 points)  Some of the time□ (4 points)  Rarely (5 points)  Never (6 points) | | | | |  |
| 6b.Feeling energized? | All the time□ (1 point)  Most of the time□ (2 points)  A lot of time□ (3 points)  Some of the time□ (4 points)  Rarely (5 points)  Never (6 points) | | | | |  |
| 6c.Feeling down and out? | All the time□ (1 point)  Most of the time□ (2 points)  A lot of time□ (3 points)  Some of the time□ (4 points)  Rarely (5 points)  Never (6 points) | | | | |  |
| 7. In the past 4 weeks, how much of the time did your physical health condition or emotional problems prevent you from socializing (e.g., visiting friends, family, etc.)? | All the time□ (1 point)  Most of the time□ (2 points)  A lot of time□ (3 points)  Some of the time□ (4 points)  Rarely (5 points)  Never (6 points) | | | | |  |

**Tip: If comorbidities and/or adverse events occurred between the time of signing the informed consent and enrollment, please fill in the relevant page numbers!**

**Date of visit:**

| **Date of visit：** 1st treatment | | | | | | | |
| --- | --- | --- | --- | --- | --- | --- | --- |
| Typical Symptoms | | □**_1_** Knee pain□**_2_** Morning stiffness□**_3_** Bone friction sounds□**_4_** Limited mobility  □**_5_** Floating patella test (+) (multiple choice) | | | | | |
| Push Patella Test | | □_1_Outer upper□_2_ Outer lower□_3_ Inner upper□_4_ Inner lower (multiple choice) | | | | | |
| Selection of needle entry point | | □_1_A □_2_ B □_3_C □_4_D □_5_E (Optional 1-3 needle entry points) | | | | | |
| Availability of emergency medication | | □_1_ Yes □_2_ No (Celecoxib capsules for pain NRS over 8) | | | | | |
| **Date of visit：** 2nd treatment | | | | | | | |
| Typical Symptoms | | □**_1_** Knee pain□**_2_** Morning stiffness□**_3_** Bone friction sounds□**_4_** Limited mobility  □**_5_** Floating patella test (+) (multiple choice) | | | | | |
| Push Patella Test | | □_1_Outer upper□_2_ Outer lower□_3_ Inner upper□_4_ Inner lower (multiple choice) | | | | | |
| Selection of needle entry point | | □_1_A □_2_ B □_3_C □_4_D □_5_E (Optional 1-3 needle entry points) | | | | | |
| Availability of emergency medication | | □_1_ Yes □_2_ No (Celecoxib capsules for pain NRS over 8) | | | | | |
| **Date of visit：** 3rd treatment | | | | | | | |
| Typical Symptoms | | □**_1_** Knee pain□**_2_** Morning stiffness□**_3_** Bone friction sounds□**_4_** Limited mobility  □**_5_** Floating patella test (+) (multiple choice) | | | | | |
| Push Patella Test | | □_1_Outer upper□_2_ Outer lower□_3_ Inner upper□_4_ Inner lower (multiple choice) | | | | | |
| Selection of needle entry point | | □_1_A □_2_ B □_3_C □_4_D □_5_E (Optional 1-3 needle entry points) | | | | | |
| Availability of emergency medication | | □_1_ Yes □_2_ No (Celecoxib capsules for pain NRS over 8) | | | | | |
| **Date of visit：** 4th treatment | | | | | | | |
| Typical Symptoms | | □**_1_** Knee pain□**_2_** Morning stiffness□**_3_** Bone friction sounds□**_4_** Limited mobility  □**_5_** Floating patella test (+) (multiple choice) | | | | | |
| Push Patella Test | | □_1_Outer upper□_2_ Outer lower□_3_ Inner upper□_4_ Inner lower (multiple choice) | | | | | |
| Selection of needle entry point | | □_1_A □_2_ B □_3_C □_4_D □_5_E (Optional 1-3 needle entry points) | | | | | |
| Availability of emergency medication | | □_1_ Yes □_2_ No (Celecoxib capsules for pain NRS over 8) | | | | | |
| **访视日期：** 5th treatment | | | | | | | |
| Typical Symptoms | | □**_1_** Knee pain□**_2_** Morning stiffness□**_3_** Bone friction sounds□**_4_** Limited mobility  □**_5_** Floating patella test (+) (multiple choice) | | | | | |
| Push Patella Test | | □_1_Outer upper□_2_ Outer lower□_3_ Inner upper□_4_ Inner lower (multiple choice) | | | | | |
| Selection of needle entry point | | □_1_A □_2_ B □_3_C □_4_D □_5_E (Optional 1-3 needle entry points) | | | | | |
| Availability of emergency medication | | □_1_ Yes □_2_ No (Celecoxib capsules for pain NRS over 8) | | | | | |
| **Date of visit：** 6th treatment | | | | | | | |
| Typical Symptoms | | □**_1_** Knee pain□**_2_** Morning stiffness□**_3_** Bone friction sounds□**_4_** Limited mobility  □**_5_** Floating patella test (+) (multiple choice) | | | | | |
| Push Patella Test | | □_1_Outer upper□_2_ Outer lower□_3_ Inner upper□_4_ Inner lower (multiple choice) | | | | | |
| Selection of needle entry point | | □_1_A □_2_ B □_3_C □_4_D □_5_E (Optional 1-3 needle entry points) | | | | | |
| Availability of emergency medication | | □_1_ Yes □_2_ No (Celecoxib capsules for pain NRS over 8) | | | | | |
| **Date of visit：** 7th treatment | | | | | | | |
| Typical Symptoms | | □**_1_** Knee pain□**_2_** Morning stiffness□**_3_** Bone friction sounds□**_4_** Limited mobility  □**_5_** Floating patella test (+) (multiple choice) | | | | | |
| Push Patella Test | | □_1_Outer upper□_2_ Outer lower□_3_ Inner upper□_4_ Inner lower (multiple choice) | | | | | |
| Selection of needle entry point | | □_1_A □_2_ B □_3_C □_4_D □_5_E (Optional 1-3 needle entry points) | | | | | |
| Availability of emergency medication | | □_1_ Yes □_2_ No (Celecoxib capsules for pain NRS over 8) | | | | | |
| **Date of visit：** 8th treatment | | | | | | | |
| Typical Symptoms | | □**_1_** Knee pain□**_2_** Morning stiffness□**_3_** Bone friction sounds□**_4_** Limited mobility  □**_5_** Floating patella test (+) (multiple choice) | | | | | |
| Push Patella Test | | □_1_Outer upper□_2_ Outer lower□_3_ Inner upper□_4_ Inner lower (multiple choice) | | | | | |
| Selection of needle entry point | | □_1_A □_2_ B □_3_C □_4_D □_5_E (Optional 1-3 needle entry points) | | | | | |
| Availability of emergency medication | | □_1_ Yes □_2_ No (Celecoxib capsules for pain NRS over 8) | | | | | |
| **Date of visit：** 9th treatment | | | | | | | |
| Typical Symptoms | | □**_1_** Knee pain□**_2_** Morning stiffness□**_3_** Bone friction sounds□**_4_** Limited mobility  □**_5_** Floating patella test (+) (multiple choice) | | | | | |
| Push Patella Test | | □_1_Outer upper□_2_ Outer lower□_3_ Inner upper□_4_ Inner lower (multiple choice) | | | | | |
| Selection of needle entry point | | □_1_A □_2_ B □_3_C □_4_D □_5_E (Optional 1-3 needle entry points) | | | | | |
| Availability of emergency medication | | □_1_ Yes □_2_ No (Celecoxib capsules for pain NRS over 8) | | | | | |
| **Date of visit：** 10th treatment | | | | | | | |
| Typical Symptoms | | □**_1_** Knee pain□**_2_** Morning stiffness□**_3_** Bone friction sounds□**_4_** Limited mobility  □**_5_** Floating patella test (+) (multiple choice) | | | | | |
| Push Patella Test | | □_1_Outer upper□_2_ Outer lower□_3_ Inner upper□_4_ Inner lower (multiple choice) | | | | | |
| Selection of needle entry point | | □_1_A □_2_ B □_3_C □_4_D □_5_E (Optional 1-3 needle entry points) | | | | | |
| Availability of emergency medication | | □_1_ Yes □_2_ No (Celecoxib capsules for pain NRS over 8) | | | | | |
| **Date of visit：** 11th treatment | | | | | | | |
| Typical Symptoms | | □**_1_** Knee pain□**_2_** Morning stiffness□**_3_** Bone friction sounds□**_4_** Limited mobility  □**_5_** Floating patella test (+) (multiple choice) | | | | | |
| Push Patella Test | | □_1_Outer upper□_2_ Outer lower□_3_ Inner upper□_4_ Inner lower (multiple choice) | | | | | |
| Selection of needle entry point | | □_1_A □_2_ B □_3_C □_4_D □_5_E (Optional 1-3 needle entry points) | | | | | |
| Availability of emergency medication | | □_1_ Yes □_2_ No (Celecoxib capsules for pain NRS over 8) | | | | | |
| **Date of visit：** 12th treatment | | | | | | | |
| Typical Symptoms | | □**_1_** Knee pain□**_2_** Morning stiffness□**_3_** Bone friction sounds□**_4_** Limited mobility  □**_5_** Floating patella test (+) (multiple choice) | | | | | |
| Push Patella Test | | □_1_Outer upper□_2_ Outer lower□_3_ Inner upper□_4_ Inner lower (multiple choice) | | | | | |
| Selection of needle entry point | | □_1_A □_2_ B □_3_C □_4_D □_5_E (Optional 1-3 needle entry points) | | | | | |
| Availability of emergency medication | | □_1_ Yes □_2_ No (Celecoxib capsules for pain NRS over 8)  28 | | | | | |
| **Drug treatment** | | | | | | | |
| **Name of drug** | **dosages** | | **unit of dose** | **frequency of administration** | **route of administration** |  |  |
|  |  |  |  |  |  | **Start date** | **End date** |
| **Celecoxib Capsules** |  | |  |  |  |  |  |
| **Celecoxib Capsules** |  | |  |  |  |  |  |
| **Celecoxib Capsules** |  | |  |  |  |  |  |
| **Celecoxib Capsules** |  | |  |  |  |  |  |
| **Celecoxib Capsules** |  | |  |  |  |  |  |
| **Celecoxib Capsules** |  | |  |  |  |  |  |
| **Celecoxib Capsules** |  | |  |  |  |  |  |

| **Adverse event：**Were there any adverse events throughout the study period? □_0_ No, □_1_ Yes, if yes, please complete the table below:  (Use standard medical terminology) Record all observations and use the following question, “How do you feel differently since your last treatment?” Ask directly about the resulting adverse event. Try to use the name of the diagnosis rather than the name of the symptom. Record one adverse event per column. | | | | | | | | | | | | |
| --- | --- | --- | --- | --- | --- | --- | --- | --- | --- | --- | --- | --- |
| **serial number** | **Description of Adverse Events** | **Start date** | **Was it sustained at the end of the study?** | | **level of severity** | **Relevance to FSN treatment** | **Measures taken for treatment** | **Other measures adopted** | **vest** | | **是否SAE** | |
|  |  |  | **Yes** | **No→Mitigation/end date** |  |  |  |  |  |  | **否** | **是，SAE具体类型**  **及报告日期** |
| **1** |  |  | □**_1_** |  | □**_1_** mild  □**_2_** moderately  □**_3_** severe | \|__\| | \|__\| | \|__\| | \|__\| | | □**_0_** | \|__\|， |
| **2** |  |  | □**_1_** |  | □**_1_** mild  □**_2_** moderately  □**_3_** severe | \|__\| | \|__\| | \|__\| | \|__\| | | □**_0_** | \|__\|， |
| **3** |  |  | □**_1_** |  | □**_1_** mild  □**_2_** moderately  □**_3_** severe | \|__\| | \|__\| | \|__\| | \|__\| | | □**_0_** | \|__\|， |
| **4** |  |  | □**_1_** |  | □**_1_** mild  □**_2_** moderately  □**_3_** severe | \|__\| | \|__\| | \|__\| | \|__\| | | □**_0_** | \|__\|， |
| **5** |  |  | □**_1_** |  | □**_1_** mild  □**_2_** moderately  □**_3_** severe | \|__\| | \|__\| | \|__\| | \|__\| | | □**_0_** | \|__\|， |
| **6** |  |  | □**_1_** |  | □**_1_** mild  □**_2_** moderately  □**_3_** severe | \|__\| | \|__\| | \|__\| | \|__\| | | □**_0_** | \|__\|， |
| **7** |  |  | □**_1_** |  | □**_1_** mild  □**_2_** moderately  □**_3_** severe | \|__\| | \|__\| | \|__\| | \|__\| | | □**_0_** | \|__\|， |
| **8** |  |  | □**_1_** |  | □**_1_** mild  □**_2_** moderately  □**_3_** severe | \|__\| | \|__\| | \|__\| | \|__\| | | □**_0_** | \|__\|， |
| **9** |  |  | □**_1_** |  | □**_1_** mild  □**_2_** moderately  □**_3_** severe | \|__\| | \|__\| | \|__\| | \|__\| | | □**_0_** | \|__\|， |
| **10** |  |  | □**_1_** |  | □**_1_** mild  □**_2_** moderately  □**_3_** severe | \|__\| | \|__\| | \|__\| | \|__\| | | □**_0_** | \|__\|， |
| **11** |  |  | □**_1_** |  | □**_1_** mild  □**_2_** moderately  □**_3_** severe | \|__\| | \|__\| | \|__\| | \|__\| | | □**_0_** | \|__\|， |
| **12** |  |  | □**_1_** |  | □**_1_** mild  □**_2_** moderately  □**_3_** severe | \|__\| | \|__\| | \|__\| | \|__\| | | □**_0_** | \|__\|， |
| **Correlation of adverse events with treatment**  1=Certainly relevant  2=Likely to be relevant  3=Likely to be relevant  4=Unlikely to be relevant  5=To be evaluated  6=Unable to evaluate | | **Measures taken for treatment**  1=no change  2=Reduce frequency/reduce amount of stimulation  3=Suspend treatment  4=Permanent cessation of treatment | | **Other measures adopted**  1=None  2=Hospitalization or extended stay, complete SAE form  3=Combination of medications, detail at Combination of Medications  4=Other, please describe | | | **Regression of AE**  1=Recovery, without sequelae  2=Recovery with sequelae  3=Relief  4=Persisting/no significant change  5=Aggravated/worsened  6=Death  7=Unknown | | | **Specific types of SAE**  1=causes death  2=Life-threatening  3=Leads to hospitalization or prolonged hospitalization  4=Leads to permanent or significant disability/loss of function  5=Led to congenital malformation  6=Other significant medical event | | |

**Severity: Mild - usually transient and does not interfere with normal daily activities; Moderate - considerable discomfort that interferes with normal daily activities, subject's self-awareness of symptoms is obvious but tolerable, no need to stop treatment; Severe --Severe - inability to carry out normal daily activities, subject's self-perceived symptoms are significant and intolerable, need to stop treatment.**

24

| Patient self-exclusion |
| --- |
| Choose only one of the following two  □**_1_**Subjects completed this study（Date of completion： ）  □**_2_**Subjects withdrew from this study（Date of withdrawal： ） |
| **If the subject withdraws from the trial, select one of the following primary reasons for withdrawal：** |
| □**_1_**Does not meet inclusion/exclusion criteria, please specify：  □**_2_**Adverse incident, number\|___\|___\|（Adverse event form completed）  □**_3_**Although no adverse events occurred, the investigators concluded that termination of treatment was in the best interest of the subjects for safety reasons  □**_4_**Patient self-exclusion  □**_5_**Patient lost visits  □**_6_**Other reasons (e.g. pregnancy, death)： |

| **Case Report Form (CRF) Audit Statement** |
| --- |
| **(Please confirm and sign each of the following checklists)** |
| 1. Confirm that the subject has signed the informed consent form. |
| 2. Confirm that the subject's name, mailing address, telephone number, etc. are filled in true and complete. |
| 3. Confirm that the subject meets the inclusion criteria of the trial program and does not meet the exclusion criteria. |
| 4. Confirm that the treatment records of the subject's group are correct. |
| 5. Confirm that all items in the study record are filled out completely. |
| 6. Confirm that the Adverse Event Form has been completed for all adverse events. Adverse events, as well as pre-treatment normal, post-treatment abnormal physical and chemical data that cannot be explained by deterioration of the disease have been reviewed and followed up to normal. |
| 7.Confirm that the “Reason for Shedding” form has been filled out for withdrawal and loss of visits. |
| 8. When it was confirmed that there was an error, the incorrect value was marked with “-”, the correct value was written above the error, the person who made the correction signed and dated it, the reason for the change was stated, and no original data were overwritten. |
| Signature of the researcher：____________ Date of signature：  Signature of evaluator：____________ Date of signature：  Signature of the quality control officer：____________ Date of signature： |

| **Principal Investigator Statement** |
| --- |
| I have reviewed all records on this case report form, page by page and item by item, and I confirm that these data are filled out truthfully, completely and accurately, are consistent with the original source material, and are in accordance with the study protocol design. All data recording was done by me and my delegate and we have signed the researcher signature form.  Signature of the principal investigator：____________  Date of signature： |
