# Supplementary material for: Fu’s subcutaneous needling approach versus electroacupuncture for knee osteoarthritis: protocol for comparative effectiveness and safety randomized controlled trial
Source: Front Med (Lausanne). 2025 May 29;12:1510326. doi: 10.3389/fmed.2025.1510326 (PMC12159060; doi:10.3389/fmed.2025.1510326)
Supplement: Supplementary file 2 [file Table_1.doc]

|  | **STUDY PERIOD** | | | | | | |
| --- | --- | --- | --- | --- | --- | --- | --- |
|  | **Screening** | **Baseline** | **Treatment** | | | | **Follow- up** |
| **TIME POINT** | (-T1) | (T0) | (T1) | (T2) | (T3) | (T4) | (T8) |
|  | **Basic information collection** | | | | | | |
| Eligibility screen | × |  |  |  |  |  |  |
| Informed consent | × |  |  |  |  |  |  |
| Allocation |  | × |  |  |  |  |  |
| demographic data |  | × |  |  |  |  |  |
| Medical and treatment history |  | × |  |  |  |  |  |
| Knee joint X-ray |  | × |  |  |  |  |  |
|  | **Safety observation** | | | | | | |
| Adverse event |  |  | × | × | × | × |  |
|  | **Therapeutic observation** | | | | | | |
| level walking（NRS） |  | × | × | × | × | × | × |
| stair activity（NRS） |  | × | × | × | × | × | × |
| 6-minute walk test |  | × | × | × | × | × | × |
| WOMAC |  | × | × | × | × | × | × |
| SF-12 |  | × |  |  |  | × | × |
| **Notes: -T1: the day before enrolment. T0: the day of enrolment and allocation. T1–T4: the treatment period for 4 weeks. T8: the Fourth week after the end of treatment.**  **NRS, Numerical Rating Scale ;WOMAC，the Western Ontario and Mc Master University Osteoarthritis Index;SF-12，Quality of Life Evaluation Scale.** | | | | | | | |

| **Acupoint** | **Acupoint number** | **Acupoint position** |
| --- | --- | --- |
| **Required Acupoints** | | |
| Dubi | ST35 | In the anterior knee region, in the lateral depression of the patellar ligament |
| Neixiyan | EX | At the knee, in the center of the medial depression of the patellar ligament |
| Ququan | LR8 | In the knee, at the inner end of the popliteal crease, in the concave inner edge of the semitendinosus tendon |
| Xiyangguan | GB33 | In the depression between the knee, the posterior upper edge of the lateral epicondyle of the femur, and the tendon of the biceps femoris muscle and the iliotibial tract |
| Ashixue |  | Pain is the acupoint, and if there is pain, it is the acupoint |
| **Optional Acupoints** | | |
| Futu | ST32 | At the anterior femoral region, six inches above the patellar floor, at the line connecting the anterior superior iliac spine and the lateral end of the patellar floor. |
| Liangqiu | ST34 | In the anterior femoral area, 2 inches above the patellar floor, between the lateral femoral muscle and the tendon of the rectus femoris muscle |
| Zusanli | ST36 | On the outer side of the calf, 3 inches below the calf's nose, 1 transverse finger outside the anterior tibial spine |
| Fenglong | ST40 | On the outer side of the calf, 8 inches above the tip of the outer ankle, at the outer edge of the anterior tibial muscle, at a transverse finger on the outer side of the Tiaokou |
| Heding | EX | In the anterior knee region, in the depression above the midpoint of the patellar floor |
| Xuhai | SP10 | In the anterior femoral area, 2 inches above the medial end of the patella, at the bulging point of the medial femoral muscle |
| Yinlingquan | SP9 | In the depression between the medial condylar edge of the tibia and the medial edge of the tibia on the inner side of the calf |
| Yingu | KI10 | On the medial side of the popliteal fossa, when the knee is flexed, when the semitendinosus tendon is between the semitendinosus tendon and the semimembranosus tendon. |
| Sanyinjiao | SP6 | On the inner side of the calf, 3 inches above the tip of the inner ankle, at the posterior edge of the medial edge of the tibia |
| Taixi | KI3 | In the ankle area, the depression between the inner ankle tip and the Achilles’tendon |
| Fengshi | GB31 | In the femur, 7 inches above the patellar floor: with the hand upright and the palm pressed against the thigh, the depression indicated by the middle finger tip and the posterior edge of the iliotibial tract |
| Yanglingquan | GB34 | On the outer side of the calf, in the depression below the anterior part of the fibular head |
| Waiqiu | GB36 | On the outer side of the calf, 7 inches above the outer ankle tip, at the anterior edge of the fibula |
| Xuanzhong | GB39 | On the outer side of the calf, 3 inches above the outer ankle tip, at the anterior edge of the fibula |
| Zulinqi | GB41 | On the dorsum of the foot, in front of the junction of the 4th and 5th metatarsal bones, in the lateral depression of the extensor digitorum longus tendon of the 5th toe |
| Weiyang | BL39 | At the knee, along the popliteal crease, at the inner edge of the biceps femoris tendon |
| Weizhong | BL40 | In the posterior knee area, at the midpoint of the popliteal crease |
| Chengshan | BL57 | At the intersection of the gastrocnemius abdomens and tendons in the posterior region of the calf |
| Kunlun | BL60 | In the depression between the outer ankle tip and the Achilles tendon in the ankle area |
